# Multilevel Stressors and Systemic and Tumor Immunity in Black and White Women With Breast Cancer

**DOI:** 10.1001/jamanetworkopen.2024.59754

**Published:** 2025-02-14

**Authors:** Alexandra R. Harris, Catherine M. Pichardo, Jamirra Franklin, Huaitian Liu, William Wooten, Gatikrushna Panigrahi, Wayne R. Lawrence, Margaret S. Pichardo, Brittany D. Jenkins, Tiffany H. Dorsey, Olga B. Ioffe, Harry G. Yfantis, Tanya Agurs-Collins, Stefan Ambs

**Affiliations:** 1 Laboratory of Human Carcinogenesis, Center for Cancer Research, National Cancer Institute, National Institutes of Health, Bethesda, Maryland; 2 Division of Cancer Control and Population Sciences, National Cancer Institute, National Institutes of Health, Rockville, Maryland; 3 University of Maryland Marlene and Stewart Greenebaum Comprehensive Cancer Center Biostatistics Shared Service, Baltimore; 4 Metabolic Epidemiology Branch, Division of Cancer Epidemiology and Genetics, National Cancer Institute, National Institutes of Health, Rockville, Maryland; 5 Department of Surgery, Hospital of the University of Pennsylvania, Philadelphia; 6 Department of Biochemistry and Molecular Biology, Johns Hopkins Bloomberg School of Public Health, Baltimore, Maryland; 7 Department of Pathology, University of Maryland School of Medicine, Baltimore; 8 Pathology and Laboratory Medicine, Baltimore Veterans Affairs Medical Center, Baltimore, Maryland

## Abstract

**Importance:**

The mechanisms through which chronic stressors may be associated with tumor biologic characteristics, immune response, and health disparities remain insufficiently understood.

**Objective:**

To investigate the proteomic, transcriptomic, and genomic effects associated with multilevel chronic stressors (perceived stress, perceived inadequate social support, perceived racial and ethnic discrimination, and neighborhood deprivation) in Black and White women with breast cancer.

**Design, Setting, and Participants:**

This cross-sectional study was conducted from February 28, 2012, to September 5, 2023, in which blood samples, breast tumors, and adjacent noncancerous tissues were collected from women with breast cancer. Participants, recruited at 2 Baltimore, Maryland, hospitals, completed demographic and psychosocial questionnaires. Data analysis was conducted from September 2023 to April 2024.

**Exposures:**

Perceived stress, perceived social support, perceived racial and ethnic discrimination, and the 2010 census tract-level neighborhood deprivation index, in which scores range from −2.51 to 6.77, with higher scores indicating greater deprivation.

**Main Outcomes and Measures:**

The main outcomes included levels of 92 circulating immune-oncologic markers and associated biologic pathways, tumor immune cell profiles, breast tissue gene expression, and tumor mutational burden. Data were analyzed using covariate-adjusted linear regression modeling for continuous outcomes with effect estimates presented as β values with 95% CIs.

**Results:**

The study included 121 women with breast cancer (mean [SD] age, 56.27 [12.62] years), of whom 56 (46.3%) were Black, and 65 (53.7%) were White. The analytic subsample sizes included 117 blood samples, 48 breast tumors, and 41 adjacent noncancerous tissues. Levels of perceived stress and social support were comparable by race, while Black women resided in more socioeconomically deprived neighborhoods (mean [SD] neighborhood deprivation index, 2.28 [2.30] for Black women compared with −0.22 [2.01] for White women). Greater perceived social support was associated with more favorable immune-stimulatory changes (eg, increased serum IL-5 [β, 0.06 (95% CI, 0.02-0.10); *P* = .003] and activated natural killer cells in noncancerous breast tissue of Black women [β, 0.11 (95% CI, 0.04-0.17); *P* = .002). Higher levels of perceived stress, exposure to discrimination, and neighborhood deprivation were associated with systemic inflammation (eg, serum IL-6 with both perceived stress [β, 0.04 (95% CI, 0.01-0.07); *P* = .006] and discrimination [β, 0.69 (95% CI, 0.15-1.23); *P* = .01]); deleterious immune cell profiles (eg, tumor-associated M2 macrophages with discrimination [β, 0.82 (95% CI, 0.14-1.51); *P* = .02]); and aggressive tumor biologic characteristics. Race-stratified analyses uncovered distinct immunologic features in Black women associated with stressors, including chemotaxis with stress (β, 0.28 [95% CI, 0.001-0.56]; *P* = .049) and immune suppression with stress (β, 0.37 [95% CI, −0.002 to 0.75]; *P* = .05) at the systemic level and increased tumor-associated myeloid cells (monocytes and M1 and M2 macrophages) at the tissue level. Perceived stress was associated with elevated tumor mutational burden (β, 0.02 [95% CI, 0.01-0.04]; *P* = .04).

**Conclusions and Relevance:**

The findings of this cross-sectional study of Black and White women with breast cancer suggest that perceived stress, perceived inadequate social support, perceived racial and ethnic discrimination, and neighborhood deprivation were associated with deleterious alterations to the systemic and tumor immune environment, particularly for Black women. Understanding biology as a possible mediator of cancer health disparities may inform prevention and public health interventions.

## Key Points

**Question:**

How are stress-related factors associated with circulating immune-inflammation markers, immune profiles, and tumor biologic characteristics in women with breast cancer?

**Findings:**

In this cross-sectional study of 121 Black and White women with breast cancer, perceived stress, perceived inadequate social support, perceived racial and ethnic discrimination, and neighborhood deprivation were associated with suppression of the antitumor immune response, systemic inflammation, and deleterious tumor biologic characteristics. These outcomes were particularly pronounced in Black women.

**Meaning:**

These findings suggest that chronic stressor-induced inflammation and immune dysfunction may be associated with increased breast cancer aggressiveness and cancer disparities in socially vulnerable and minoritized populations.

## Teaser

This cross-sectional study of Black and White women with breast cancer evaluates whether multilevel stressors are associated with tumor biologic characteristics, an immune response, and exacerbated health disparities.

## Introduction

Black women endure excessive breast cancer mortality.^1^ Chronic stress and social isolation increase cancer metastasis and may negatively impact breast cancer survival and exacerbate health disparities.^2,3,4,5,6^ Furthermore, structural racism and discrimination are candidate root causes for cancer disparities among Black individuals.^7,8^ At the interpersonal level, racial and ethnic discrimination is a chronic stressor. It may prompt individuals to remain in a state of rumination, worry, and vigilance.^9^ At the structural level, Black women are more likely to reside in segregated communities with greater neighborhood deprivation (eg, concentrated poverty); environmental hazards; and unequal access to adequate health care, healthy foods, and physical activity opportunities.^6,10,11^ Exposure to these types of chronic stressors can increase breast cancer mortality.^6,12,13,14,15^

Chronic stress activates biologic signaling pathways (eg, epigenetic modifications, immune regulation or activation, and angiogenesis) associated with DNA damage and aging, which are relevant to cancer.^16,17,18,19^ Yet, because a biologic link between psychosocial stressors and cancer outcomes has not been fully elucidated, current clinical standards of care do not routinely incorporate interventions to address stress responses and improve cancer survival in patients.^16^ Furthermore, limited research has examined the biologic mechanisms through which multilevel stressors (eg, chronic stress, inadequate social support, interpersonal racial and ethnic discrimination, and neighborhood deprivation) may influence cancer outcomes.^6,16^ Thus, there is a need to identify how stress-related social determinants may be associated with tumor biologic characteristics in patients with cancer to inform clinical management. This study addresses these gaps in knowledge by examining multilevel chronic stressors and their association with the systemic and local immune environment at the proteomic, transcriptomic, and genomic level within Black and White women with breast cancer.

## Methods

### Study Population

This cross-sectional study included 217 self-reported Black or African American (hereinafter referred to as Black) and White women with breast cancer (aged 28 to 90 years) undergoing breast cancer surgery between February 28, 2012, and September 5, 2023, who were recruited from 2 hospitals in Baltimore, Maryland, (the University of Maryland Medical Center and the University of Maryland Baltimore Washington Medical Center) into the National Cancer Institute–Maryland Breast Cancer Stress Study. Additional details of the study population and design are provided in the eMethods in Supplement 1. The study was approved by the University of Maryland institutional review board. All participants signed a written informed consent. This study followed the Strengthening the Reporting of Observational Studies in Epidemiology (STROBE) reporting guideline.

### Exposures and Covariates

#### Perceived Stress

Perceived stress (hereinafter referred to as stress) was estimated with the Cohen 10-item Perceived Stress Scale,^20^ which captures an individual’s appraisal of potentially stressful situations. Scores can range from 0 to 40, with higher scores indicating greater stress. See the eMethods in Supplement 1 for more details.

#### Perceived Social Support

Perceived social support (hereinafter referred to as social support) was measured using the 24-item Social Provisions Scale.^21^ The Social Provisions Scale captures 6 relational provisions, including guidance, reliable alliance, reassurance of worth, social integration, attachment, and opportunity to provide nurturance. Scores can range from 24 to 96, with higher scores indicating greater social support. See the eMethods in Supplement 1 for more details.

#### Perceived Racial and Ethnic Discrimination and Related Symptoms

Perceived racial and ethnic discrimination and related symptoms (hereinafter referred to as discrimination) were measured using 3 items from the Reaction to Race scale used in the Behavioral Risk Factor Surveillance System.^22^ A composite score was created and dichotomized as 0, indicating no experiences of discrimination (experiences of equal or better race-based treatment) or related physical or emotional symptoms, and 1, indicating experiences of discrimination or related symptoms. See the eMethods in Supplement 1 for more details.

#### Neighborhood Deprivation

Census tract-level neighborhood deprivation was measured using the Neighborhood Deprivation Index, which summarizes 6 census tract-level variables (percentage of households in poverty, female-headed households with dependent children, households on public assistance, households earning less than $30 000 per year, unemployed individuals, and manager occupation) from 2010 into a single standardized index for statistical analyses, as previously described.^23^ Scores range from −2.51 to 6.77, with higher scores indicating greater deprivation. See the eMethods in Supplement 1 for more details.

#### Covariates

Covariates included body mass index, age, socioeconomic status (composite score, accounting for both educational and income levels), and race (Black or White). The tumor subtype was not correlated with any exposure. See the eMethods in Supplement 1 for more details.

### Serum Proteomic Profiling

Our group previously described the method for measuring the 92 immune-oncologic markers in human serum samples using Olink technology.^24^ These circulating proteins were grouped into 6 pathways (eTable 1 in Supplement 1). See the eMethods in Supplement 1 for more details.

### Gene Expression, Immune Cell Deconvolution, and Tumor Mutational Burden

Total RNA was isolated from frozen breast tissue and sequenced as previously described by our group.^25^ Immune cell deconvolution was performed using CIBERSORTx.^26^ See the eMethods in Supplement 1 for more details. Tumor mutational burden (TMB) in breast tumors was assessed from whole-exome sequence data through the summation of all nonsynonymous somatic sequence variations, as described by our group.^25^

### Statistical Analysis

Data analysis was conducted from September 2023 to April 2024. Additional stratification by ethnicity was not performed due to low numbers of Hispanic or Latina participants (eMethods in Supplement 1). Associations between exposures were examined and are shown in eTable 2 in Supplement 1. Covariate-adjusted linear regression modeling was performed for all continuous outcomes (levels of circulating immune-oncologic markers, serum proteomics-defined biologic pathway scores, immune cell subpopulation abundance, and TMB [log-transformed]), with effect estimates presented as β coefficients with 95% CIs. Statistical significance was defined as a 2-sided *P* < .05 for immune-oncologic marker and pathway models. A 2-sided *P* ≤ .10 or a false discovery rate (FDR) of less than 10% was used as the cutoff to report an association for transcriptomic and genomic analyses. See the eMethods in Supplement 1 for more details. Analyses were performed using Stata/SE, version 17.0 (StataCorp LLC) and RStudio statistical software, version 2021.09.0.351 (Posit Software, PBS).

## Results

### Participant Characteristics

The analytic subsample sizes for each study aim were 117 proteomic samples (circulating immune-oncologic markers and serum proteomics-defined biologic pathway analyses), 48 tumor and 41 tumor-adjacent noncancerous (normal) gene-expression profiling samples, and 46 tumor whole-exome sequencing samples (TMB analyses) (see eFigure 1 in Supplement 1 for the overlap of samples across study aims). Details of the study population of 121 women with breast cancer (mean [SD] age, 56.27 [12.62] years), of whom 56 (46.3%) were Black and 65 (53.7%) were White, can be found in eResults and eTables 3 and 4 in Supplement 1. Levels of stress and social support were comparable by race, but Black women resided in more socioeconomically deprived neighborhoods (mean [SD] neighborhood deprivation index, 2.28 [2.30] for Black women and −0.22 [2.01] for White women). No significant correlations were observed between exposures, except for discrimination and social support (*r* = 0.43; *P* = .003) (eTable 2 in Supplement 1).

### Stress-Related Determinants and Circulating Immune-Oncologic Markers

**Figure 1.**
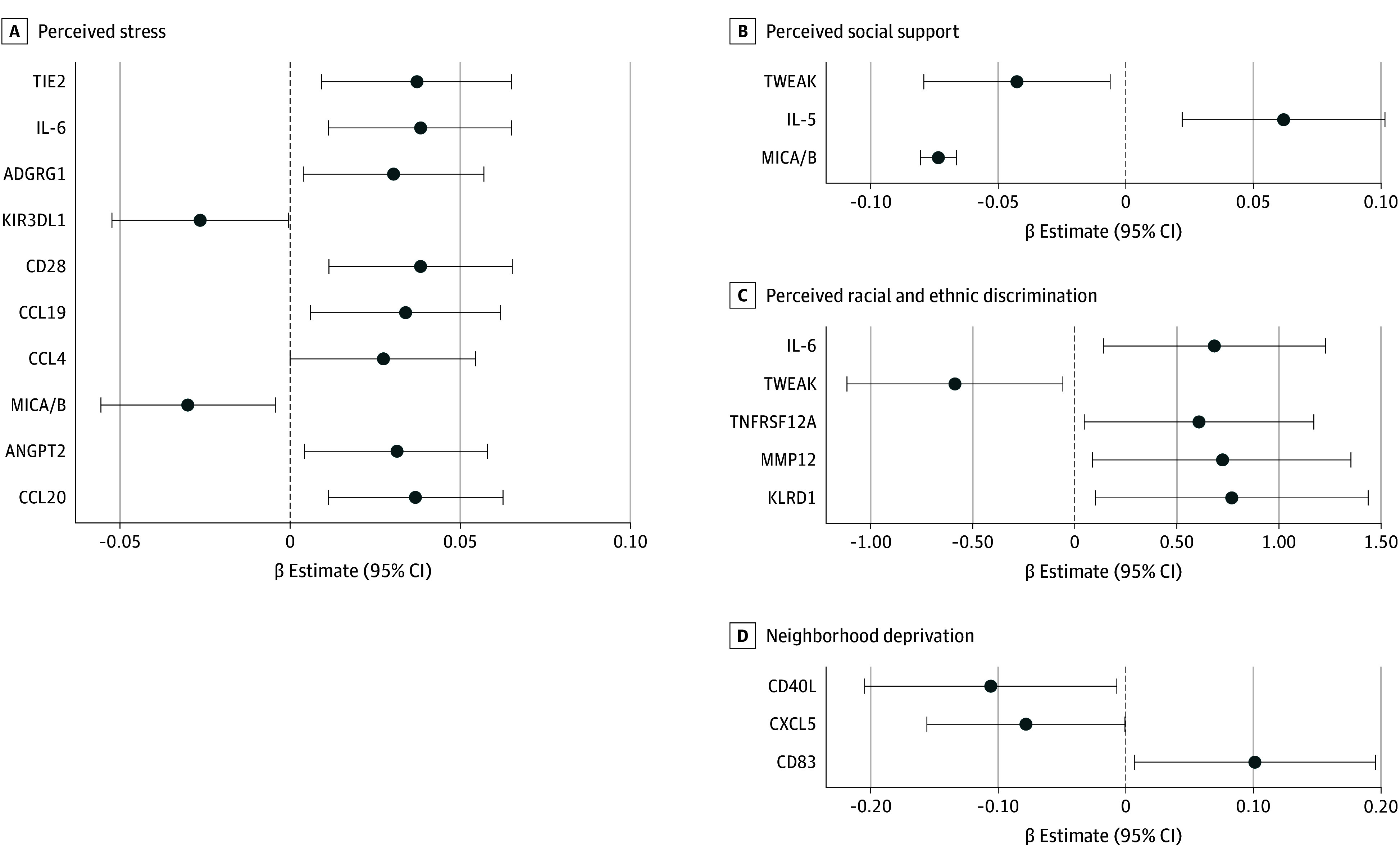
Association of Perceived Stress, Perceived Social Support, Perceived Racial and Ethnic Discrimination, and Neighborhood Deprivation With Circulating Immune-Oncologic Markers β Estimates and 95% CIs from linear regression of each exposure associated with immune-oncologic markers among Black and White women with a breast cancer diagnosis is shown (*P* < .05). Only significant markers are included in plots. Separate models adjusted for age at study entry (continuous), body mass index at study entry (continuous), self-reported race (Black and White, not included in the racial and ethnic discrimination model), and socioeconomic status (ie, high school or less and in poverty, at least some college and in poverty, high school or less and not in poverty, and at least some college and not in poverty). Poverty was defined using household income and the US federally defined poverty threshold based on household size, age, and recruitment year. Perceived stress (n = 117) (A), perceived social support (n = 117) (B), and neighborhood deprivation (n = 111) (D) models were conducted among the full sample. The perceived racial and ethnic discrimination model was conducted only among Black women (n = 43) (C). ADGRG1 indicates adhesion G protein-coupled receptor G1; ANGPT2, angiopoietin 2; CXCL, C-X-C motif chemokine ligand; IL, interleukin; KIR3DL1, killer cell immunoglobulin like receptor, 3 Ig domains and long cytoplasmic tail 1; KLRD1, killer cell lectin like receptor D1; MIC, MHC class I polypeptide-related sequence; MMP12: matrix metallopeptidase 12; TIE2, tyrosine kinase with immunoglobulin like and EGF like domains 2; TNFRSF12A, TNF receptor superfamily member 12A; and TWEAK, TNF superfamily member 12.

Serum levels of 10 circulating immune-oncologic markers were associated with stress, 3 with social support, 5 with discrimination, and 3 with neighborhood deprivation in covariate-adjusted analyses (Figure 1 and eTables 9-12 in Supplement 2). Stress was associated with elevated levels of several chemokines known to promote immune cell recruitment and cancer cell invasion (CCL4: β, 0.03 [95% CI, 0.0002-0.05], *P* = .048; CCL19: β, 0.03 [95% CI, 0.01-0.06], *P* = .02; and CCL20: β, 0.04 [95% CI, 0.01-0.06], *P* = .005), as well as proangiogenic factors (TIE2: β, 0.04 [95% CI, 0.01-0.07]; *P* = .009 and ANGPT2: β, 0.03 [95% CI, 0.005-0.06]; *P* = .02) and the proinflammatory cytokine IL-6 (β, 0.04 [95% CI, 0.01-0.07]; *P* = .006) (Figure 1A). We also observed associations between stress and proteins that may suggest diminished presence and activity of natural killer (NK) cells, including reduced circulating levels of the NK receptor KIR3DL1 (β, −0.03 [95% CI, −0.05 to −0.0003]; *P* = .047) and the NK activator MICA/B (β, −0.03 [95% CI, −0.06 to −0.004]; *P* = .02) and increased levels of the NK inhibitory receptor ADGRG1 (β, 0.03 [95% CI, 0.004-0.06]; *P* = .02). Higher levels of social support, which can buffer against the otherwise negative impact of stress and discrimination on health, was associated with increased IL-5 (β, 0.06 [95% CI, 0.02-0.10]; *P* = .003), a cytokine produced primarily by T cells to boost immune function (Figure 1B). Discrimination was positively associated with IL-6 (β, 0.69 [95% CI, 0.15-1.23]; *P* = .01) and TNFRSF12A (β, 0.61 [95% CI, 0.05-1.17]; *P* = .03), another inflammation-related factor (Figure 1C). In addition, MMP12 (β, 0.72 [95% CI, 0.09-1.35]; *P* = .03), a macrophage-secreted matrix metalloproteinase known to promote breast cancer progression, was elevated in Black women experiencing discrimination. Higher neighborhood deprivation was associated with increased circulating levels of soluble CD83 (β, 0.10 [95% CI, 0.01-0.20]; *P* = .03) (Figure 1D), which has been shown to be associated with inhibition of T-cell function.^27,28^

**Figure 2.**
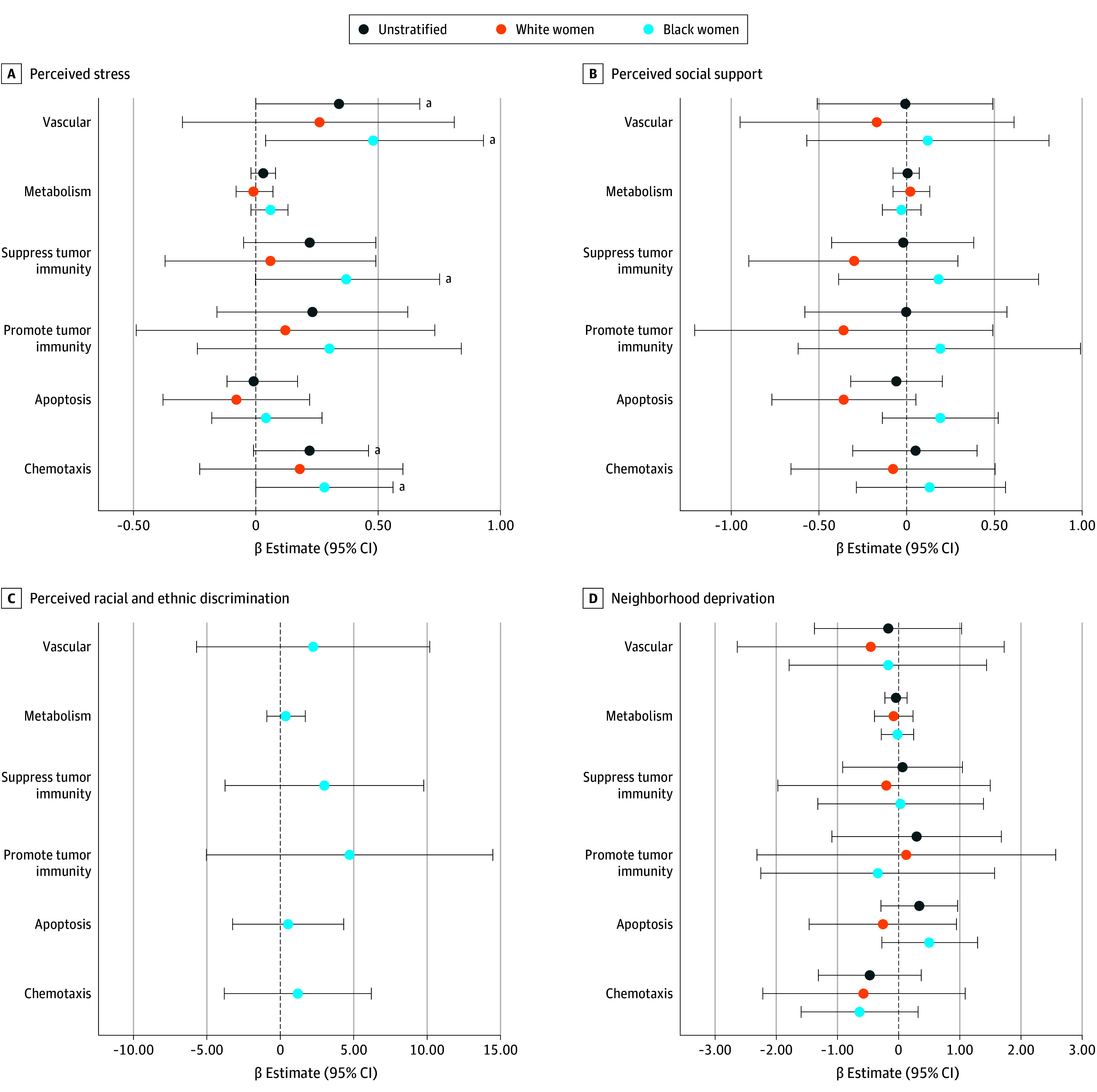
Association of Perceived Stress, Perceived Social Support, Perceived Racial and Ethnic Discrimination, and Neighborhood Deprivation With Serum Proteomics-Defined Biologic Pathways β Estimates and 95% CIs from linear regression of each exposure associated with immune-oncologic pathways among Black and White women with a breast cancer diagnosis is shown. Separate models adjusted for age at study entry (continuous), body mass index at study entry (continuous), self-reported race (Black and White, not included in the racial and ethnic discrimination model), and socioeconomic status (ie, high school or less and in poverty, at least some college and in poverty, high school or less and not in poverty, and at least some college and not in poverty). Poverty was defined using household income and the US federally defined poverty threshold based on household size, age, and recruitment year. Perceived stress (n = 117 [Black women, n = 52; White women, n = 65]) (A), perceived social support (n = 117 [Black women, n = 52; White women, n = 65]) (B), and neighborhood deprivation (n = 111 [Black women, n = 50; White women, n = 61]) (D) models were conducted among the full sample. The perceived racial and ethnic discrimination model was conducted only among Black women (n = 43). ^a^*P* < .10.

We next evaluated associations of the stress-related determinants with the activity scores of 6 serum proteomic-defined biologic pathways (eTable 1 in Supplement 1). In unstratified analyses, only stress showed an association with pathway activities (Figure 2 and eTable 5 in Supplement 1), namely with angiogenesis and vascular remodeling (β, 0.34 [95% CI, −0.0006 to 0.67]; *P* = .05) and chemotaxis (β, 0.22 [95% CI, −0.01 to 0.46]; *P* = .06), which reached significance among Black women (angiogenesis: β, 0.48 [95% CI, 0.04-0.93]; *P* = .03; chemotaxis: β, 0.28 [95% CI, 0.001-0.56]; *P* = .049). We also observed an association between stress and suppression of antitumor immunity (β, 0.37 [95% CI, −0.002 to 0.75]; *P* = .05) among Black women. We next examined models that investigated the association of stress and proteomic-defined biologic pathways with all stress-related exposures in the same model, using a stepwise approach among Black and White women combined. Adjustment for neighborhood deprivation attenuated the association between stress and biologic pathways, indicating that neighborhood deprivation may play a role in the association between stress and pathway activities (eTable 6 in Supplement 1).

### Stress-Related Determinants and the Breast Immune Microenvironment

Given that chronic stressors were associated with changes to the systemic immune microenvironment, we hypothesized that stress-related determinants may alter the local tumor-immune microenvironment within breast tissue. In covariate-adjusted analyses within a subset of participants with matched transcriptomic data (eTable 4 in Supplement 1), several notable associations between stress-related exposures and the cellular composition of the local immune microenvironment were uncovered, which were generally more pronounced in the noncancerous tissues (Table 1).

**Table 1.** Immune Cell Profiles in Tumor and Tumor-Adjacent Normal Tissues Associated With Multilevel Stress-Related Determinants^a^

Immune cell populations	Perceived stress, β			Perceived social support, β			Perceived discrimination for Black women, β^b^	Neighborhood deprivation, β		
	Unstratified	Black women	White women	Unstratified	Black women	White women		Unstratified	Black women	White women
**Tumor**										
Naive B cells	−0.01	−0.02	−0.03	0	−0.001	−0.004	0.29	−0.04	−0.09	−0.06
Plasma cells	−0.01	−0.02	−0.02	−0.004	0.01	−0.04	0.26	−0.13^c^	−0.20^d^	−0.15
CD8 T cells	0.01	−0.001	−0.02	0.01	0.03	−0.04	0.69^c^	0.08	−0.04	0.35^d^
CD4 memory resting T cells	−0.03	−0.05^c^	0.09^e^	0.02	−0.004	0.09^d^	−0.54	0.01	0.05	−0.07
Follicular helper T cells	−0.04^e^	−0.03	−0.02	−0.04^c^	−0.07^e^	0.01	−0.38	−0.13^c^	−0.06	−0.03
Tregs	0.01	0.02	−0.06	−0.03	−0.03	−0.03	−0.21	0.11	0.11^c^	−0.14
NK cells, resting	0.02	−0.02	0.18^f^	0.04^c^	0.02	0.09	−0.04	−0.02	−0.11^c^	−0.06
NK cells, activated	−0.04^c^	−0.03	−0.01	0.004	−0.01	0.04	0.15	−0.03	0.07	−0.10
Monocytes	0.01	−0.001	0.05	0.01	0.02	0.03	0.51	0.02	0.02	0.04
M0 macrophages	−0.003	0.03	−0.06	−0.02	−0.03	−0.04	−0.56	0.03	0.01	0.10
M1 macrophages	0.01	−0.02	0.03	−0.01	0.004	−0.05	0.61^c^	0.09	0.12	−0.06
M2 macrophages	0.04^d^	0.05^d^	0.02	0.01	0.05^c^	−0.03	0.48	0.01	0.09	0.01
Dendritic cells, activated	0.02	0.03	−0.04	0.03	0.02	0.04	0.45	−0.08	−0.17^c^	0.16
Mast cells, resting	−0.02	−0.01	−0.04	0.01	0.01	0.03	0.12	0.04	0.05	0.05
**Noncancerous breast tissue**										
Naive B cells	−0.03	−0.03^c^	−0.06	−0.04	−0.04	−0.11^c^	−0.21	0.28^f^	0.23^e^	0.35
Plasma cells	−0.02	−0.02	0.01	−0.06^e^	−0.06^e^	−0.03	−0.56^c^	0.05	0.07	0.11
CD8 T cells	−0.02	−0.04	0.03	−0.03	−0.03	−0.03	−0.70^c^	0.11	0.03	0.54^d^
CD4 memory resting T cells	0.02	0.04	−0.08	0.04	0.09^e^	−0.08	0.73^c^	−0.17^d^	−0.14	−0.15
Follicular helper T cells	−0.07^f^	−0.07^f^	−0.07^c^	−0.02	−0.02	−0.09^e^	−0.96^e^	0.23^f^	0.21^d^	0.17
CD4 naive T cells	0.03^c^	0.004	0.13^d^	0.04^c^	0.03	0.10^c^	0.30	−0.14^d^	−0.17^e^	−0.02
NK cells, resting	0.04^c^	0.03	0.08	0.03	0.01	0.06	0.82	−0.09	−0.11	−0.21
NK cells, activated	−0.02	−0.01	−0.08^d^	0.06^e^	0.11^f^	−0.09^e^	0.34	−0.01	−0.02	−0.01
Monocytes	0.03	0.05^d^	0.07	0.05^c^	0.04	0.07	0.33	−0.22^e^	−0.21^e^	−0.13
M0 macrophages	−0.01	−0.004	−0.12^e^	−0.03	−0.02	−0.09^c^	−0.48	0.23^e^	0.25^e^	0.02
M1 macrophages	0.01	−0.001	−0.03	0.01	0.01	−0.01	0.58	0.13^c^	0.24^f^	−0.79^f^
M2 macrophages	0.04^c^	0.03^c^	0.09	0.04^c^	0.01	0.18^f^	0.82^e^	−0.21^e^	−0.19^e^	−0.24
Dendritic cells, activated	−0.01	−0.002	0.02	−0.01	−0.004	−0.05	−0.28	0.13^c^	0.05	0.40
Mast cells, resting	0.01	0.01	0.06	−0.03	−0.05^c^	0.08	−0.59	−0.15^c^	−0.10	−0.21

Abbreviation: NK, natural killer.

^a^ All models were adjusted for the following confounders, selected a priori: age at diagnosis, body mass index, socioeconomic status (composite), and race (with the exception of discrimination models in Black women only).

^b^ Discrimination models were conducted only among Black women.

^c^ *P* < .20.

^d^ *P* < .10.

^e^ *P* < .05.

^f^ *P* < .001.

In both tumor and tumor-adjacent noncancerous tissues, higher stress was associated with decreased abundance of follicular helper T cells (tumor: β, −0.04 [95% CI, −0.08 to −0.0003]; *P* = .049; noncancerous tissue: β, −0.07 [95% CI, −0.11 to −0.03]; *P* = .002), a type of CD4 T cell associated with better breast cancer prognosis, and increased levels of immunosuppressive tumor-associated M2 macrophages (tumor: β, 0.04 [95% CI, −0.001 to 0.09]; *P* = .053). These immune profiles are indicative of a tumor-promoting local microenvironment. Higher levels of social support were associated with decreased levels of follicular helper T cells in tumor tissues of Black women (β, −0.07 [95% CI, −0.12 to −0.006]; *P* = .03) and noncancerous tissues of White women (β, −0.09 [95% CI, −0.17 to −0.003]; *P* = .045). While higher levels of social support among White women were associated with decreased levels of activated NK cells (β, −0.09 [95% CI, −0.17 to −0.01]; *P* = .03) in noncancerous tissue, the opposite was true for Black women (β, 0.11 [95% CI, 0.04-0.17]; *P* = .002), suggesting differential effects by race. Similar to stress, discrimination was also associated with decreased levels of follicular helper T cells (β, −0.96 [95% CI, −1.83 to −0.09]; *P* = .03) and increased levels of immunosuppressive M2 macrophages (β, 0.82 [95% CI, 0.14-1.51]; *P* = .02) in noncancerous tissues. Lastly, higher neighborhood deprivation was associated with increased numbers of naive B cells, follicular helper T cells, and M0 and M1 macrophages, as well as decreased numbers of CD4 memory resting T cells, CD4 naive T cells, monocytes, M2 macrophages, and resting mast cells within noncancerous tissue. The association between neighborhood deprivation and proinflammatory macrophages differed by race (Black women: β, 0.24 [95% CI, 0.07-0.42]; *P* = .01; White women: β, −0.79 [95% CI, −1.24 to −0.34]; *P* = .005).

### Stress-Related Determinants and Breast Tumor Biologic Characteristics

We next examined whether stress-related determinants were associated with tumor biologic characteristics (ie, gene expression), adjusting for potential confounders. The 5 top-ranked differentially expressed protein-coding genes associated with stress-related determinants in both noncancerous and cancerous tissues are displayed in eTables 7 and 8 in Supplement 1; full gene lists can be found in eTables 13 to 20 in Supplement 2. Stress was associated with 68 differentially expressed genes (DEGs); social support, 54 DEGs; discrimination, 902 DEGs; and neighborhood deprivation, 33 DEGs, in a consistent manner across noncancerous and cancerous tissues (FDR <0.1). To identify the key signaling pathways in which these genes were involved, an overrepresentation analysis was performed using the significantly expressed DEGs in the tumor, which found that stress was associated with upregulation of 6 and downregulation of 0 pathways, social support with upregulation and downregulation of 0 pathways, discrimination with upregulation of 15 and downregulation of 10 pathways, and neighborhood deprivation with upregulation of 3 and downregulation of 0 pathways in breast tumors (FDR <0.1) (Table 2 and eTables 7 and 8 in Supplement 1).

**Table 2.** Enriched Tumor Signaling Pathways Associated With Multilevel Stress-Related Determinants^a^

Pathway	Activated signaling pathways in tumor, adjusted *P* value^b^
Perceived stress	
Dopamine receptors	.09
RUNX1 regulates transcription of genes involved in BCR signaling	.09
Immunoregulatory interactions between a lymphoid and a nonlymphoid cell	.09
Immune system	.09
Endosomal or vacuolar pathway	.09
IL-27 signaling	.09
Perceived social support	NA
Perceived discrimination^c^	
Endosomal or vacuolar pathway	<.001
IFN-γ response	.002
Allograft rejection	.002
IL-6/JAK/STAT3 signaling	.002
Hypoxia	.004
Antigen presentation: folding, assembly, and peptide loading of MHC class I	.01
IFN-α/β signaling	.01
Cytokine signaling in immune system	.04
Antigen processing cross presentation	.07
TNF-α signaling via NFκB	.01
UV response up	.03
Complement	.03
Bile acid metabolism	.07
IL-2/STAT5 signaling	.07
KRAS signaling up	.07
Neighborhood deprivation	
Gap junction assembly	.049
Gap junction trafficking	.049
Gap junction trafficking and regulation	.049

Abbreviations: BCR, B-cell antigen receptor; IFN, interferon; IL, interleukin; JAK, janus kinase; MHC, major histocompatibility complex; NA, not applicable; NFκB, nuclear factor-κB; RUNX1, runt-related transcription factor 1; STAT, signal transducer and activator of transcription; TNF, tumor necrosis factor; UV, ultraviolet.

^a^ All models were adjusted for the following confounders, selected a priori: age at diagnosis, body mass index, socioeconomic status (composite), and race (with the exception of discrimination models in Black women only).

^b^ Based on significantly differentially overexpressed genes (false discovery rate <0.1). Reactome and Molecular Signatures Database cancer hallmark pathways.

^c^ Discrimination models were conducted only among Black women.

Most of the top-ranked DEGs upregulated with stress were immune related. In noncancerous tissue, top DEGs included chemokine *CCL18* (fold change [FC] = 6.54; FDR = 6.36 × 10^−2^), which is released by tumor-associated macrophages to promote cancer cell invasion, and coagulation factor *F8A3* (FC = 3.18; FDR = 1.36 × 10^−4^). In tumors, top DEGs included *HLA-G* (FC = 18.13; FDR = 1.72 × 10^−2^), which is known to promote tumor immune evasion, and complement receptor *CR2* (FC = 16.45; FDR = 4.15 × 10^−2^). Stress showed significant enrichment for 6 pathways involved not only in immune function and inflammation but also in the stress-induced catecholamine signaling pathway.

Social support was associated with upregulation of several immunoglobulin G–related DEGs in both noncancerous (*IGLV3-19*: FC = 11.71; FDR = 9.75 × 10^−2^) and tumor (*IGKV1-33*: FC = 66.72; FDR = 3.01 × 10^−3^) tissues; immunoglobulin expression within breast tumors can be favorable (indicator of B-cell activity)^29^ or detrimental (tumor cell derived, protumorigenic),^30^ depending on the source of their secretion. Other interesting factors emerged, such as *ADAMDEC1* (FC = 8.23; FDR = 9.75 × 10^−2^), whose expression was shown to be associated with increased chemosensitivity and an improved prognosis in patients with breast cancer, and gap junctional protein GJB5, which is generally considered tumor suppressive.^31^ We observed no significant pathway enrichment in association with social support.

Discrimination in Black women showed the largest number of exposure-associated DEGs among the 4 stress-related exposures. Of the 902 discrimination-associated DEGs, 45 (5.0%) were shared between noncancerous tissue and tumor expression profiles. In noncancerous tissue, the top DEGs included stearoyl-coenzyme A desaturase (FC = 7.36; FDR = 1.55 × 10^−2^), with a known role in lipid reprogramming to promote cancer cell growth, and oxidative stress-induced neuronatin (FC = 4.72; FDR = 9.84 × 10^2^). In tumors, the top DEGs included growth differentiation factor 5 (FC = 89.88; FDR = 1.62 × 10^−4^), which regulates transforming growth factor-β–dependent angiogenesis in breast cancer, and interestingly, the spexin hormone (FC = 84.45; FDR = 5.11 × 10^−3^), which is regulated by serotonin and upregulated during times of social stress in animal models.^32^ Of the 15 discrimination-associated pathways with enrichment patterns in breast tumors, 10 (66.7%) were associated with immune response and inflammatory signaling, including antigen presentation (MHC class I) and proinflammatory cytokine signaling (IFN-α/β, TNF-α, IL-2, IL-6, and IFN-γ). Similar to stress, we observed enrichment for endosomal pathways, whose dysregulation in tumors has been previously associated with progression. Notably, proliferative signaling through KRAS and hypoxia signaling were also significantly enriched in Black women experiencing discrimination.

Fewer significant DEGs emerged that were associated with neighborhood deprivation (total, 33 [tumor-adjacent normal, 11; tumor, 22]). The top 3 most significant upregulated DEGs (*CCL18*, *DNAH10*, and *F8A3*) in noncancerous tissues overlapped with what we observed with stress, suggesting that the biologic consequences of stress on the tumor may be partially agnostic to the stressor itself, whether at the individual or neighborhood level. In tumor tissues, DEGs such as *GJB5* (FC = 30.19; FDR = 9.48 × 10^−2^), a connexin with a known role in cancer progression, and melanotransferrin/*CD228* (FC = 4.57; FDR = 9.85 × 10^−2^), a cancer-specific protein with increased expression in several types of solid tumors, were also upregulated in women residing in more socioeconomically deprived neighborhoods. While only 3 pathways were significantly enriched in association with neighborhood deprivation, all were associated with gap junctions. Gap junctional signaling is a stress-regulated component of catecholamine release,^33^ and its dysregulation can promote cancer cell invasion.^34^

### Stress-Related Determinants and TMB

Within a subset of women with matched whole-exome sequence data, we tested for associations between each of the multilevel stress-related determinants and TMB, adjusting for potential confounders. While no significant associations were observed with most stressors, a higher level of stress was associated with a higher TMB (β, 0.02 [95% CI, 0.01-0.04]; *P* = .04) (eFigure 2 in Supplement 1).

## Discussion

In this cross-sectional study, we assessed the association of multilevel stress-related determinants with the systemic immune environment, the local tumor microenvironment, and tumor biologic characteristics using proteomic, transcriptomic, and genomic data. We identified novel, distinct molecular features associated with each stress-related factor, as well as common pathways shared across them (summarized in Table 3 and eFigure 3 in Supplement 1).

**Table 3.** Summary of Key Biologic Outcomes of Stress-Related Determinants at the Genomic, Transcriptomic, and Proteomic Level

Stress-related determinant	Tumor genome	Tumor transcriptome	Local immune microenvironment	Systemic immune microenvironment
Perceived stress	Increased tumor mutational burden	DEGs: 68 (eg, *CCL18*, *F8A3*, *HLA-G)* Enriched pathways: 6 (eg, inflammation, cytokine signaling, catecholamine signaling, endosomal)	Decreased follicular helper T cells Increased M2 macrophages (mostly in Black women) Increased monocytes (in Black women only) Increased resting NK cells (in White women only)	Circulating protein markers: 10 (eg, increase in chemokines [CCL4, CCL19, CCL20]) Higher inflammatory cytokine IL-6 Increase in proangiogenic (TIE2, ANGPT2) NK cell-related (KIR3DL1, MICA/B, ADGRG1) Biologic pathways: Increase in angiogenesis and vascular remodeling Increase in chemotaxis Increased suppression of antitumor immunity (in Black women only)
Perceived social support	No significant association	DEGs: 54 (eg, *IGLV3-19*, *IGKV1-33*, *ADAMDEC1*) Enriched pathways: 0	Increased CD4 memory resting T cells Decreased plasma cells Activated NK cells: Increased in Black women Decreased in White women Decreased follicular helper T cells, increased M2 macrophages (in White women only)	Circulating protein markers: 3 (eg, increase in immune-boosting IL-5)
Perceived discrimination^a^	No significant association	DEGs: 902 (eg, *SCD*, *NNAT*, *SPX*, *GDF5*) Enriched pathways: 15 (eg, endosomal, proinflammatory cytokines, proliferation, immune response, hypoxia)	Decreased follicular helper T cells Increased M2 macrophages	Circulating protein markers: 3 (eg, increase in inflammation [IL-6, TNFRSF12A], proinvasion MMP12)
Neighborhood deprivation	No significant association	DEGs: 33 (eg, *CCL18*, *DNAH10*, *F8A3*, *GOLGA6B*) Enriched pathways: 3 (eg, dysregulation of gap junctions)	Increased M0 macrophages Increased naive B cells and follicular helper T cells Decreased CD4 memory resting T cells, CD4 naive T cells, monocytes, M2 macrophages M1 macrophages: Increased in Black women Decreased in White women	Circulating protein markers: 5 (eg, higher levels of CD83)

Abbreviations: DEGs, differentially expressed genes; NK, natural killer.

^a^ Black women only.

Systemically, stress was associated with circulating proteins known to promote immune and cancer cell chemotaxis, angiogenesis, and inflammation, most robustly in Black women. The positive association between stress and suppression of anti-tumor immunity was most apparent in Black women. In the local immune microenvironment, stress was associated with fewer follicular helper T cells, reduced NK activity, and increased immunosuppressive M2 macrophages; immune cell profiles were generally more deleterious in Black women. Stress was associated with enrichment of immune, inflammatory, and catecholamine signaling pathways and increased TMB, a known stimulator of tumor immunogenicity,^35^ in tumors. When taken together, these data suggest that prolonged stress in patients with breast cancer may lead to an inflammatory systemic and local immune environment, suppressing an antitumor immune response and exacerbating tumor progression. These findings are consistent with the literature on the potential impact of stress on the body and tumor biologic characteristics.^18,36,37^ Yet, while evidence for these connections has been strong in preclinical animal models,^38^ data on patients are still sparse. These findings may contribute to translating preclinical findings to patients.

Inadequate social support is a chronic stressor^39^ and associated with cancer mortality.^40,41,42^ Animal studies report that social experiences can also directly impact physiology by elevating stress hormones.^43,44,45^ However, studies investigating how social support may affect tumor biologic characteristics in patients with cancer are needed.^6^ While we did not observe a marked association of social support with tumor biologic characteristics, our study showed immune modulatory outcomes at local and systemic levels that indicate tumor-suppressing outcomes, most favorably in Black women.

Racism is associated with worse cancer outcomes in Black individuals.^46,47^ A prior study suggested that discrimination experiences were associated with accelerated aging and the inflammatory markers C-reactive protein and IL-6^48^; yet, there remains a paucity of research linking racism and discrimination to tumor biologic characteristics.^6^ In this study, our measure of racial and ethnic discrimination captured both the experiences of discrimination and the physical and emotional symptoms associated with these experiences. We uncovered a transcriptome of over 900 discrimination-associated genes and enrichment in 15 discrimination-associated signaling pathways, of which most are related to immune response, inflammation, and other protumorigenic processes such as hypoxia, proliferation, and endosomal activity. Our observations suggest that the resulting biologic consequences of discrimination may be similar to those of stress in their association with inflammation and immunosuppression.

Understanding how neighborhoods and other structural factors may impact cancer risk and outcomes through biologic mechanisms is a high-priority area of health disparities research.^49^ A deprived neighborhood environment was shown to be associated with inflammation^37^ through increased levels of C-reactive protein and IL-6,^50,51,52^ allostatic load and accelerated biologic aging,^53^ epigenomic modifications,^54^ and neutrophil activity.^54^ At the systemic level, deprivation was associated with increased circulating levels of soluble CD83, which has been shown to inhibit T-cell function.^27,28^ Although neighborhood deprivation was not associated directly with TMB, we observed a deprivation-associated gene expression pattern that overlapped with that of stress; this is not unexpected, as residing in socioeconomically deprived areas in itself can induce psychological distress.^55^ Overall, the neighborhood-level environment showed similar and distinct biologic outcomes compared with individual-level stressors.

### Strengths and Limitations

This study includes an assessment of stress-related exposures at multiple levels, which is a strength. We included important confounders, such as socioeconomic status, in our analyses. The large proportion of Black women (46.3%) also represents a strength, as Black individuals experience a disproportionate burden of stressors across multiple levels.

Our study also has limitations, including cohort size. Cross-sectional study designs do not allow for causal inference. The study was geographically restricted to Baltimore and surrounding areas in Maryland. We did not possess residential history data and were thus unable to capture neighborhood deprivation longitudinally. While we used single-item measures of discrimination and related symptoms, we addressed this limitation by creating a composite score.^56^ The Perceived Stress Scale measures stress after a diagnosis of breast cancer and may not fully capture chronic stress. Lastly, using a stepwise approach to examine the association between stress and proteomic biomarkers may have underestimated variance since sociodemographic and sociostructural variables were entered sequentially, and shared variance was attributed to these variables.^57^

## Conclusions

In this cross-sectional and hypothesis-generating study of 121 women with breast cancer, stress, inadequate social support, racial and ethnic discrimination, and neighborhood deprivation were associated with deleterious biologic and immunologic changes at the genomic, transcriptomic, and proteomic levels both systemically and within the tumor microenvironment in patients with breast cancer. Understanding biology as a possible mediator of cancer health disparities can inform prevention and public health interventions.

## Notes

Supplement 1.
eMethods.

eResults.

eReferences.
**eFigure 1.** Sample Size Across Proteomic, Transcriptomic, and Genomic Datasets **eFigure 2.** Association Between Multilevel Stress-Related Determinants and Tumor Mutational Burden **eFigure 3.** Biological Effects of Multilevel Stress-Related Determinants on the Systemic and Local Immune Microenvironment **eTable 1.** Serum Proteomics-Defined Pathways Captured by the 92 Immune-Oncology Markers **eTable 2.** Correlations Between Multilevel Stress-Related Determinants **eTable 3.** Participant Characteristics Within the Proteomic Analytic Sample **eTable 4.** Participant Characteristics Within the Tumor Transcriptomic Analytic Sample **eTable 5.** Association of Perceived Stress, Social Support, Discrimination, and Neighborhood Deprivation With Serum Proteomics-Defined Biological Pathways **eTable 6.** Associations Between Multilevel Stress-Related Determinants and Serum Proteomics-Defined Pathways Adjusted for Individual, Interpersonal, and Neighborhood-Level Factors **eTable 7.** Differential Gene Expression and Pathway Enrichment Analyses From Upregulated Genes in Relation to Multilevel Stress-Related Determinants in Tumor and Tumor-Adjacent Normal Tissues **eTable 8.** Differential Gene Expression and Pathway Enrichment Analyses From Downregulated Genes in Relation to Multilevel Stress-Related Determinants in Tumor and Tumor-Adjacent Normal Tissues

Supplement 2. **eTable 9.** Associations Between Multilevel Stress-Related Determinants and 92 Circulating Proteomic Markers: Perceived Stress as Exposure **eTable 10.** Associations Between Multilevel Stress-Related Determinants and 92 Circulating Proteomic Markers: Social Support as Exposure **eTable 11.** Associations Between Multilevel Stress-Related Determinants and 92 Circulating Proteomic Markers: Racial and Ethnic Discrimination as Exposure **eTable 12.** Associations Between Multilevel Stress-Related Determinants and 92 Circulating Proteomic Markers: Neighborhood Deprivation as Exposure **eTable 13.** Differential Gene Expression in Relation to All Multilevel Stress-Related Determinants: Stress DEGs - Tumor **eTable 14.** Differential Gene Expression in Relation to All Multilevel Stress-Related Determinants: Stress DEGs - Normal **eTable 15.** Differential Gene Expression in Relation to All Multilevel Stress-Related Determinants: Social Support DEGs - Tumor **eTable 16.** Differential Gene Expression in Relation to All Multilevel Stress-Related Determinants: Social Support DEGs - Normal **eTable 17.** Differential Gene Expression in Relation to All Multilevel Stress-Related Determinants: Discrimination DEGs - Tumor **eTable 18.** Differential Gene Expression in Relation to All Multilevel Stress-Related Determinants: Discrimination DEGs - Normal **eTable 19.** Differential Gene Expression in Relation to All Multilevel Stress-Related Determinants: NDI DEGs - Tumor **eTable 20.** Differential Gene Expression in Relation to All Multilevel Stress-Related Determinants: NDI DEGs - Normal

Supplement 3.
Data Sharing Statement
