# Supplementary material for: Multilevel Stressors and Systemic and Tumor Immunity in Black and White Women With Breast Cancer
Source: JAMA Netw Open. 2025 Feb 14;8(2):e2459754. doi: 10.1001/jamanetworkopen.2024.59754 (PMC11829235; doi:10.1001/jamanetworkopen.2024.59754)
Supplement: Supplement 1. — eMethods. eResults. eReferences. eFigure 1. Sample Size Across Proteomic, Transcriptomic, and Genomic Datasets eFigure 2. Association Between Multilevel Stress-Related Determinants and Tumor Mutational Burden eFigure 3. Biological Effects of Multilevel Stress-Related Determinants on the Systemic and Local Immune Microenvironment eTable 1. Serum Proteomics-Defined Pathways Captured by the 92 Immune-Oncology Markers eTable 2. Correlations Between Multilevel Stress-Related Determinants eTable 3. Participant Characteristics Within the Proteomic Analytic Sample eTable 4. Participant Characteristics Within the Tumor Transcriptomic Analytic Sample eTable 5. Association of Perceived Stress, Social Support, Discrimination, and Neighborhood Deprivation With Serum Proteomics-Defined Biological Pathways eTable 6. Associations Between Multilevel Stress-Related Determinants and Serum Proteomics-Defined Pathways Adjusted for Individual, Interpersonal, and Neighborhood-Level Factors eTable 7. Differential Gene Expression and Pathway Enrichment Analyses From Upregulated Genes in Relation to Multilevel Stress-Related Determinants in Tumor and Tumor-Adjacent Normal Tissues eTable 8. Differential Gene Expression and Pathway Enrichment Analyses From Downregulated Genes in Relation to Multilevel Stress-Related Determinants in Tumor and Tumor-Adjacent Normal Tissues [file jamanetwopen-e2459754-s001.pdf]

Supplemental Online Content

Harris AR, Pichardo CM, Franklin J, et al. Multilevel stressors and systemic and tumor immunity in Black and White women with breast cancer. *JAMA Netw Open*. 2025;8(2):e2459754. doi:10.1001/jamanetworkopen.2024.59754

eMethods
eResults
eReferences
eFigure 1. Sample Size Across Proteomic, Transcriptomic, and Genomic Datasets
eFigure 2. Association Between Multilevel Stress-Related Determinants and Tumor Mutational Burden
eFigure 3. Biological Effects of Multilevel Stress-Related Determinants on the Systemic and Local Immune Microenvironment
eTable 1. Serum Proteomics-Defined Pathways Captured by the 92 Immune-Oncology Markers
eTable 2. Correlations Between Multilevel Stress-Related Determinants
eTable 3. Participant Characteristics Within the Proteomic Analytic Sample
eTable 4. Participant Characteristics Within the Tumor Transcriptomic Analytic Sample
eTable 5. Association of Perceived Stress, Social Support, Discrimination, and Neighborhood Deprivation With Serum Proteomics-Defined Biological Pathways
eTable 6. Associations Between Multilevel Stress-Related Determinants and Serum Proteomics-Defined Pathways Adjusted for Individual, Interpersonal, and Neighborhood-Level Factors
eTable 7. Differential Gene Expression and Pathway Enrichment Analyses From Upregulated Genes in Relation to Multilevel Stress-Related Determinants in Tumor and Tumor-Adjacent Normal Tissues
eTable 8. Differential Gene Expression and Pathway Enrichment Analyses From Downregulated Genes in Relation to Multilevel Stress-Related Determinants in Tumor and Tumor-Adjacent Normal Tissues

This supplemental material has been provided by the authors to give readers additional information about their work.

© 2025 Harris AR et al. *JAMA Network Open*.

eMethods

Study Population. The National Cancer Institute (NCI)-Maryland Breast Cancer Stress Study. This study investigates the relationships of perceived stress, social isolation, and racial and ethnic discrimination with stress-induced signatures, prognostic gene expression signatures, and related protein markers. Briefly, 217 participants were recruited from the University of Maryland Medical Center or the Baltimore Washington Medical Center and primarily resided in the Greater Baltimore, Maryland region. Eligibility criteria included: 1) pathological diagnosis of breast cancer, 2) being female, 3) aged 30-90, 4) speaks English well enough to be interviewed, and 6) physically and mentally capable of performing the interview. Participants were excluded based on the following criteria: 1) severely ill and hospitalized in intensive care unit, 2) severe mental and physical health problems and disabilities that impeded informed consent, 3) residing in an institution, and 4) received neo-adjuvant therapy. Trained and certified study staff administered socio-demographic, behavioral, and biological assessments at time of enrollment (i.e., prior to surgery) and conducted biospecimen collection of blood (pre- and post-surgery) and both frozen and formalin-fixed paraffin-embedded (FFPE) tissue samples. Addresses were collected at enrollment and geocoded using 2010 U.S. Census Tracts Federal Information Processing Series boundaries. Participant geocodes were linked to the 2006-10 American Community Survey estimates derived from the National Neighborhood Change Database[1].

Inclusion criteria for the study. The current study includes 55 Black and 66 White women and 2 women who additionally identified as Hispanic/Latina. Recruitment efforts focused on self-identified Black and White women because the majority of women with breast cancer at the two Baltimore recruitment sites are from these two population groups. The inclusion of another population group was not advised as their sample size would have been too small for a stratified analysis. Also, Black women are twice as likely to die from breast cancer than White women in the United States. In light of these racial disparities, this study focused on Black women due to their unique experiences with historical and current day structural racism, individual discrimination, and cultural racism embedded in the socio-political context of the United States. Inclusion of White women not only provided a necessary reference population for comparison in the context of the racial disparities between Black and White women with breast cancer, but also enabled us to examine the biological impact of multi-level chronic stressors both irrespective of and stratified by race.

© 2025 Harris AR et al. *JAMA Network Open*.

Perceived stress. Perceived stress was measured with the 10-item Cohen Perceived Stress Scale (PSS-10)[2]. The PSS-10 captures an individual's appraisal of potentially stressful situations that occurred in the past month (0 = never, 1 = almost never, 2 = sometimes, 3 = fairly often, 4 = very often). Example items include: “In the last month, how often have you been upset because of something that happened unexpectedly?” and “In the last month, how often have you felt nervous and stressed?”. Responses to items that were positively worded (i.e., In the last month, how often have you felt confident about your ability to handle your personal problems?) were reverse coded. Items were summed and higher scores indicated greater perceived stress. Cronbach’s alpha revealed adequate reliability (alpha = 0.84) in this sample and was similar across patient groups (Black=0.86, White=0.81).

Perceived social support. Perceived social support was measured using the 24-item Social Provision Scale (SPS)[3]. The SPS captures six relational provisions, including guidance, reliable alliance, reassurance of worth, social integration, attachment, and opportunity to provide nurturance. Sample items included, “There are people I can depend on if I really need it” and “I feel a strong emotional bond with at least one other person.” Responses were scored on a 4-point scale ranging from 1 (strongly disagree) to 4 (strongly agree). Responses to items that were negatively worded (e.g., I do not think other people respect my skills and abilities) were reverse coded. Cronbach’s alpha revealed high reliability (alpha = 0.90) in this sample. Items were summed and higher scores indicated greater perceived social support. Scores for social support were similar across patient groups (Black=0.89, White=0.90).

Perceived racial and ethnic discrimination and related symptoms. Perceived racial and ethnic discrimination and related symptoms were assessed by asking participants to indicate if they experienced differential treatment due to their race with three items from the Reaction to Race scale used in the Behavioral Risk Factor Surveillance System[4]. The first item, discrimination, was captured with the following question: “Within the past 12 months, when seeking health care, do you feel your experiences were worse than other races (race groups: White, Black, Asian-American, Hispanic/Latino), the same as other races, better than other races, or worse than some races but better than others?”. This scale has been used in previous studies[4-6]. The remaining two items assessed the

© 2025 Harris AR et al. *JAMA Network Open*.

frequencies of emotional and physical symptoms due to race- and ethnic- based differential treatment with the following questions: “Within the past 12 months, how often have you experienced any physical symptoms, for example a headache, an upset stomach, tensing of your muscles, or a pounding heart, as a result of how you were treated based on your race?” and “Within the past 12 months, how often have you felt emotionally upset, for example angry, sad, or frustrated, as a result of how you were treated based on your race?”. The frequency scale for physical and emotional items ranged from never (1) to constantly (6). These items have been previously described[4]. Black women indicating only encountering people of the same race or not having received health care in the past year preceding the survey were excluded from analysis that examined discrimination. Following the approach of Crawford et al.[4], a composite score was created and dichotomized as 0) “no experiences of discrimination (experiences of equal or better race-based treatment) nor related physical or emotional symptoms” and 1) “experiences of discrimination or related symptoms”. Eleven participants had missing data on discrimination measures. For the study, we utilized the term discrimination to refer to both racial and ethnic interpersonal discrimination and related symptoms. We focused on the analysis of discrimination to women self-identifying as Black and/or Hispanic/Latina due to their unique experiences with historical and current day structural racism, individual discrimination, and cultural racism embedded in the socio-political context of the United States and specifically targeted at people of color[7,8].

Neighborhood deprivation. Census tract level 2010 neighborhood deprivation was measured using the Neighborhood Deprivation Index (NDI) developed by Messer and colleagues[9]. Based on prior work that validated the index in Maryland, a principal component approach was utilized to capture the shared variance of seven socio-economic neighborhood characteristics in one factor[10]. We retained six variables with a loading above > 0.25: percent households in poverty, percent female headed households with dependent children, percent households on public assistance, percent households earning under $30,000/year, percent males and females unemployed, and percent manager occupation. The index was standardized to have a mean of 0 and standard deviation of 1 and higher values indicated higher deprivation. Additional details of the index creation for the NCI-Maryland Prostate Cancer Study has been published elsewhere[11]. Six participants had missing NDI data.

© 2025 Harris AR et al. *JAMA Network Open*.

Covariates. Physiologic covariates included body mass index (BMI), which was calculated as weight (kg) divided by the square of height (m^2). Socio-demographic covariates included age at diagnosis and socio-economic status defined as 1) high school degree or less and below poverty threshold, 2) more than high school degree and below poverty threshold, 3) high school degree or less, and above poverty threshold, and 4) more than high school degree and above poverty threshold. Categorizations of education followed the approach utilized by Lewis, et al[12]. However, in deviation, to capture socio-economic status we determined poverty with the federally defined income threshold which accounts for age, household size and census year. Four study participants were missing data on socioeconomic status. Participants' race and ethnicity (White, Black/African American) was self-reported via study questionnaire. Two participants self-identified as Hispanic/Latina. Given that both Hispanic/Latina and Black populations experience multiple forms of racism, we categorized the two self-identifying Hispanic/Latina women with the Black racial group. We were not able to perform stratified analysis among the Hispanic/Latina group given the small sample size of two. We interpret race as a social construct that represents the culmination of biological, social, and environmental factors.

Serum Proteomic Profiling. Serum proteomic profiling was performed on samples from all Black/African American and White women who provided blood as part of the NCI-Maryland Breast Study (n=117). Circulating levels of 92 circulating serum proteins defined six pathway/biological process activity scores, including (1) suppression of anti-tumor immunity, (2) promotion of anti-tumor immunity, (3) chemotaxis, (4) metabolism, (5) angiogenesis/vascular remodeling, and (6) apoptosis/cell death, similar to those previously described in detail[13] (e**Table 1**). The proprietary multiplex Proximal Extension Assay by Olink Proteomics (Boston, MA) was used to measure abundance levels of the serum proteins by the service provider, Discovery Life Sciences. A sum of z-scores was calculated of associated proteins for each pathway/biological process[13], with higher scores indicating increasing pathway activity. Included proteins in this study passed a rigorous quality control of coefficients of variation among blinded duplicates of <10%. No significant differences were observed in examination of pre- and post-surgery serum protein levels. Analysis only included post-surgery serum protein measurements due to no significant differences and lower missingness when compared to pre-surgery levels. The 92 circulating proteins that we measured were grouped into these related pathways and an activity score was calculated based on protein abundance levels in the circulation, as previously described[13].

© 2025 Harris AR et al. *JAMA Network Open*.

Gene expression and immune cell profiling using RNA sequencing and CIBERSORTx. RNA isolated from 48 frozen tumors and 41 adjunct normal tissues was sent to the NCI Center for Cancer Research Sequencing Facility for library preparation with the TruSeq PolyA kit (Illumina). Sequencing was performed with the NovaSeq system using 150 bp paired-end reads with a sequence depth of at least 30 million reads. Reads were trimmed with the Trimmomatic software with 90% of them being uniquely aligned to the human genome (hg38) using STAR. RNA mapping statistics were calculated using Picard with more than 90% of the reads being mapped to the transcriptome. Read count per gene was calculated by HTSeq under the annotation of Gencode and normalized by size factor implemented in the DESeq2 package. TPM (Transcripts per million) values of gene expression were used to perform further analyses. Differential expression analysis was performed using the DESeq2 package in R, adjusting for the following potential confounders selected *a priori:* age at diagnosis, BMI at diagnosis, a composite metric to capture socioeconomic status, and race. Differentially expressed genes (DEGs) with a q value FDR<0.1 were considered significant. For pathway enrichment analysis, genes upregulated with each exposure (FDR<0.1) were then imported into the Enrichr tool[14] to perform an overrepresentation analysis of both cancer hallmark pathways (MSigDb, Broad Institute) and Reactome pathways. Pathways with an FDR<0.1 were used for subsequent biological interpretation. Immune cell deconvolution was performed using CIBERSORTx[15] and gene expression-inferred immune cell subpopulation scores were z-score transformed to improve comparability of beta estimates across cell subpopulations. Immune cell subpopulations that showed scores of zero for >80% of samples were excluded from analyses.

Tumor mutational burden (TMB). Genomic DNA was extracted from frozen breast tumors and adjacent non-cancerous tissues using the Qiagen DNeasy Blood & Tissue kit and checked for quality using the Agilent Genomic DNA Screen tape assay. Whole exome sequencing (WES) was performed by the service provider, Psomagen, and data processed as described by us[16]. TMB was determined through the summation of all nonsynonymous somatic mutations (i.e. nonstop, missense, frameshift deletions/insertions, nonsense, in-frame deletions/insertions, splice site, and translations start site mutations from each patient). TMB was calculated with a capture region of 64 MB to obtain standardized frequency estimates. TMB was log-transformed for subsequent analyses.

© 2025 Harris AR et al. *JAMA Network Open*.

Statistical Analysis. In an analysis that examined the association of stress and immune-oncology marker expression, we conducted a stepwise modeling approach. Model 1 included individual level confounders, model 2 added social support and model 3 further included the NDI. This approach allowed us to examine the unique contribution of multi-level factors. Regression models were adjusted for covariates selected *a priori*— BMI, age at diagnosis, socio-economic status, race (except for discrimination models). Tumor stage and subtype were not significantly correlated with any of our 4 exposures and were thus not included as covariates in subsequent analyses to maximize parsimony in our regression models. Point Biserial correlations revealed that discrimination was significantly related to higher levels of social support (e**Table 2**). In this study, we were solely interested in the biological impact of race-based discrimination experiences amongst Black women, thus all analyses using discrimination as the exposure were applied to Black women only. Statistical significance was defined as $P<0.05$ for immune-oncology marker and proteomics models. A two-sided $P\leq0.1$ or a false discovery rate (FDR) <10% were used as the cutoff to report an association. Analyses were performed using Stata/SE (version 17.0, Stata Corp LLC) and R/RStudio statistical software.

© 2025 Harris AR et al. *JAMA Network Open*.

eResults

Characteristics for participants whose serum underwent proteomic profiling (n=117, 56% EA, 44% AA) are shown by exposure in e**Table 3**. Characteristics for a subset of participants whose tumor tissue underwent transcriptomic profiling (n=48) and whole-exome sequencing (n=46) are shown by exposure in e**Table 4**. Levels of perceived stress and social support were comparable by race for both the proteomic and tumor transcriptomic samples. In contrast, Black women resided in neighborhoods with higher deprivation (proteomic samples: mean NDI (SD), Black =2.28 (2.30), White = -0.22 (2.01); tumor transcriptomic samples: mean NDI (SD), Black =2.28 (2.15), White =-0.48 (1.51)). We did not observe differences by race in perceived stress [proteomic samples: mean NDI (SD), Black = 16.33 (8.46), White = 18.28 (6.91); tumor transcriptomic samples: mean NDI (SD), Black = 16.21 (9.58), White = 20.05 (5.60)) or social support (proteomic samples: mean NDI (SD), Black = 62.27 (5.30), White = 61.32 (4.52); tumor transcriptomic samples: mean NDI (SD), Black = 63.75 (6.65), White = 61.70 (5.52)].

© 2025 Harris AR et al. *JAMA Network Open*.

eReferences

1. Geolytics. *Neighborhood change database [NCDB] tract data from 1970-2010 [Online demographic data].* 2014.

2. Cohen S, Kamarck T, Mermelstein R. A global measure of perceived stress. *J Health Soc Behav*. Dec 1983;24(4):385-96.

3. Cutrona CE, Russell DW. The provisions of social relationships and adaptation to stress. *Advances in personal relationships*. 1987;1(1):37-67.

4. Crawford ND, Jones CP, Richardson LC. Understanding the role of reactions to race-based treatment in breast and cervical cancer screening. *J Natl Med Assoc*. Feb 2008;100(2):188-96. doi:10.1016/s0027-9684(15)31207-4

5. Crawford ND, Jones CP, Richardson LC. Understanding racial and ethnic disparities in colorectal cancer screening: Behavioral Risk Factor Surveillance System, 2002 and 2004. *Ethn Dis*. Autumn 2010;20(4):359-65.

6. Purnell JQ, Peppone LJ, Alcaraz K, et al. Perceived discrimination, psychological distress, and current smoking status: results from the Behavioral Risk Factor Surveillance System Reactions to Race module, 2004-2008. *Am J Public Health*. May 2012;102(5):844-51. doi:10.2105/ajph.2012.300694

7. Williams DR, Lawrence JA, Davis BA, Vu C. Understanding how discrimination can affect health. *Health Serv Res*. Dec 2019;54 Suppl 2(Suppl 2):1374-1388. doi:10.1111/1475-6773.13222

8. Krieger N. Embodying inequality: a review of concepts, measures, and methods for studying health consequences of discrimination. *Int J Health Serv*. 1999;29(2):295-352. doi:10.2190/m11w-vwxe-kqm9-g97q

9. Messer LC, Laraia BA, Kaufman JS, et al. The development of a standardized neighborhood deprivation index. *Journal of Urban Health*. Nov 2006;83(6):1041-62. doi:10.1007/s11524-006-9094-x

10. O'Campo P, Burke JG, Culhane J, et al. Neighborhood deprivation and preterm birth among non-Hispanic Black and White women in eight geographic areas in the United States. *Am J Epidemiol*. Jan 15 2008;167(2):155-63. doi:10.1093/aje/kwm277

11. Pichardo MS, Minas TZ, Pichardo CM, et al. Association of Neighborhood Deprivation With Prostate Cancer and Immune Markers in African American and European American Men. *JAMA Netw Open*. Jan 3 2023;6(1):e2251745. doi:10.1001/jamanetworkopen.2022.51745

© 2025 Harris AR et al. *JAMA Network Open*.

12. Lewis MW, Khodneva Y, Redmond N, et al. The impact of the combination of income and education on the incidence of coronary heart disease in the prospective Reasons for Geographic and Racial Differences in Stroke (REGARDS) cohort study. *BMC Public Health*. Dec 29 2015;15:1312. doi:10.1186/s12889-015-2630-4

13. Minas TZ, Candia J, Dorsey TH, et al. Serum proteomics links suppression of tumor immunity to ancestry and lethal prostate cancer. *Nature Communications*. 2022/04/01 2022;13(1):1759. doi:10.1038/s41467-022-29235-2

14. Chen EY, Tan CM, Kou Y, et al. Enrichr: interactive and collaborative HTML5 gene list enrichment analysis tool. *BMC Bioinformatics*. Apr 15 2013;14:128. doi:10.1186/1471-2105-14-128

15. Chen B, Khodadoust MS, Liu CL, Newman AM, Alizadeh AA. Profiling Tumor Infiltrating Immune Cells with CIBERSORT. *Methods Mol Biol*. 2018;1711:243-259. doi:10.1007/978-1-4939-7493-1_12

16. Tang W, Zhang F, Byun JS, et al. Population-specific Mutation Patterns in Breast Tumors from African American, European American, and Kenyan Patients. *Cancer Res Commun*. Nov 7 2023;3(11):2244-2255. doi:10.1158/2767-9764.CRC-23-0165

© 2025 Harris AR et al. *JAMA Network Open*.

eFigure 1. Sample Size Across Proteomic, Transcriptomic, and Genomic Datasets

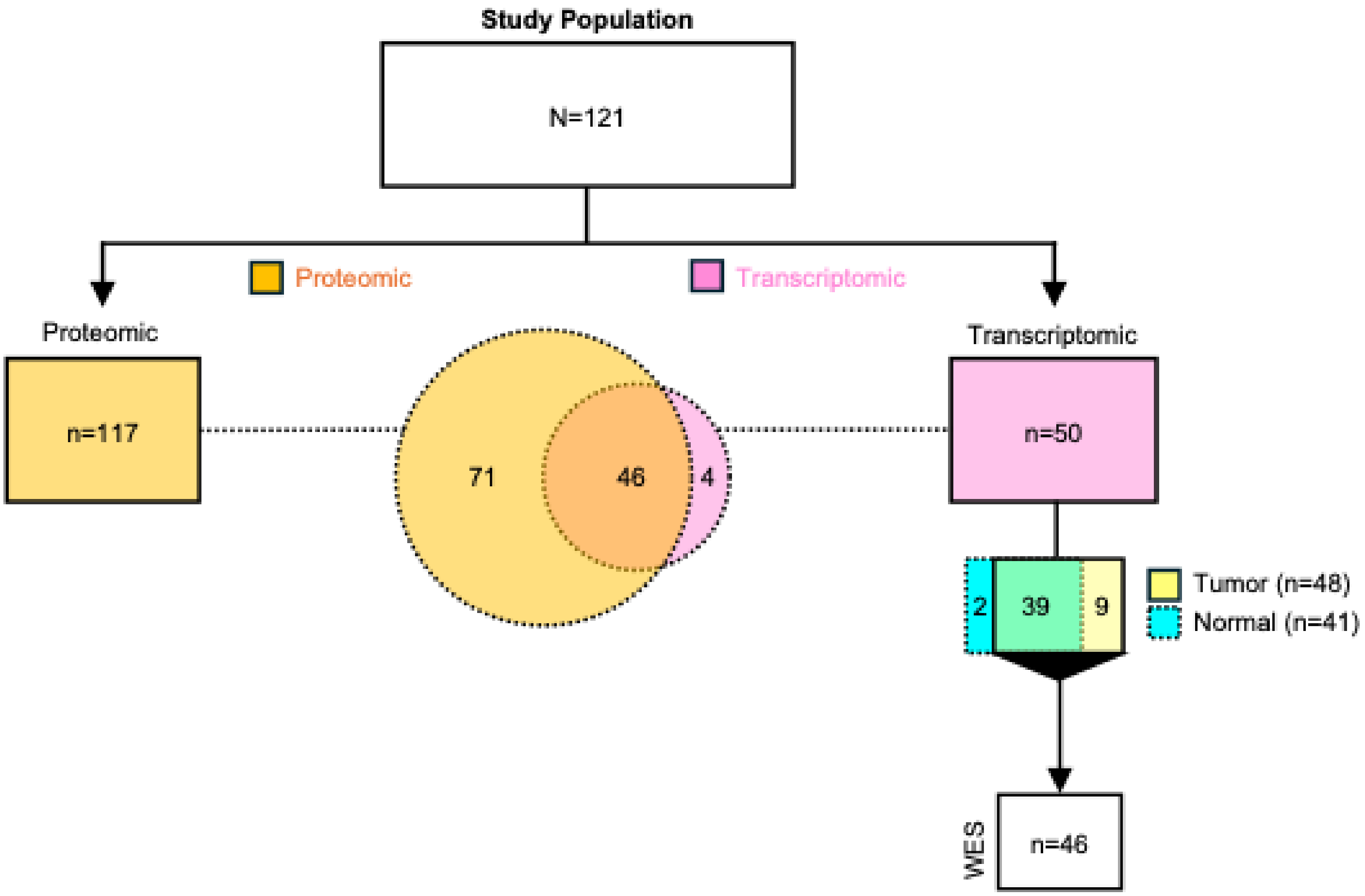

Our study included 121 participants, including 117 serum samples that underwent proteomic profiling (*orange*) and 50 tissue samples that underwent transcriptomic profiling (RNA-sequencing) (*pink*); 46 samples overlapped between proteomic and transcriptomic samples (*Venn diagram, circular*). 89 samples from 50 participants underwent RNA-sequencing (tumor=48, normal-adjacent=41); 39 of the 50 participants had matched tumor and normal-adjacent tissues (*Venn diagram, boxed*). Of the 48 tumor samples that underwent RNA-sequencing, 46 also underwent whole exome sequencing (WES), 42 of which overlapped with proteomic.

© 2025 Harris AR et al. *JAMA Network Open*.

eFigure 2. Association Between Multilevel Stress-Related Determinants and Tumor Mutational Burden

A

Associations with Tumor Mutational Burden[a,b]				
Exposure	**β**	Lower 95% CI	Upper 95% CI	*P-value*
Stress [c]	0.02	0.001	0.04	0.04
Social Support [c]	-0.001	-0.02	0.02	0.94
Discrimination [d]	-0.03	-0.43	0.36	0.85
NDI [e]	-0.04	-0.11	0.03	0.30

[a] linear regression models: TMB (tumor mutational burden, log-transformed, continuous)

[b] All models adjusted for age at diagnosis, BMI at diagnosis, socioeconomic status (composite), race (race not included in discrimination models)

[c] Perceived

[d] Discrimination models subsetted to Black women only

[e] NDI: Neighborhood deprivation index

B

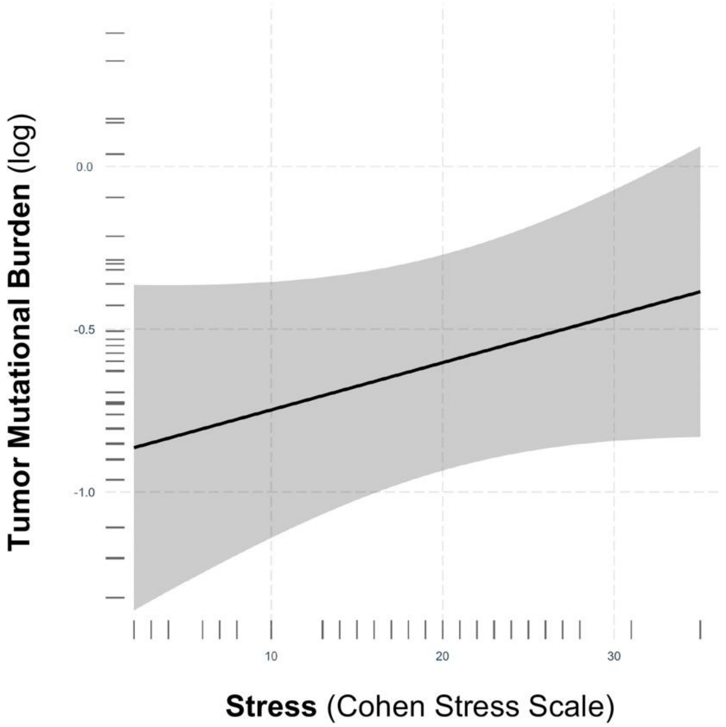

(A) Associations between each stress-related exposure and tumor mutational burden (log-transformed). Beta estimates, 95% confidence intervals (CIs), and *P* values displayed from covariate-adjusted linear regression models. Models were adjusted for age at diagnosis, body mass index at diagnosis, socioeconomic status (composite), and race, with exception of discrimination model which was subsetted to only Black women and thus not additionally adjusted for race. **(B)** Linear regression model results displayed as a rug plot to visualize predicted tumor mutational burden (log-transformed) across varying levels of perceived stress, holding all other covariates constant.

© 2025 Harris AR et al. *JAMA Network Open*.

eFigure 3. Biological Effects of Multilevel Stress-Related Determinants on the Systemic and Local Immune Microenvironment

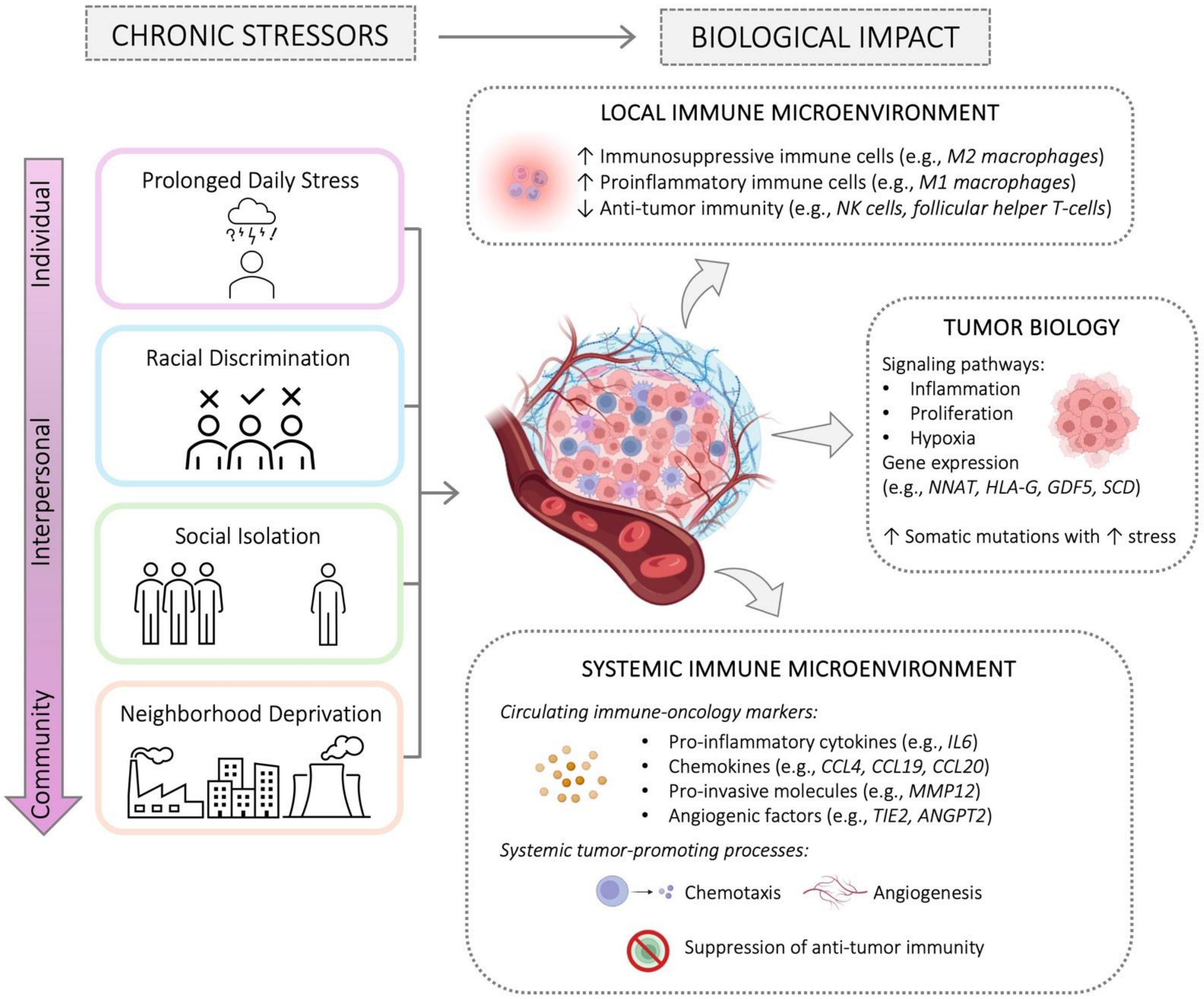

Schematic depicting key biological effects of multi-level stress-related determinants at the proteomic, transcriptomic, and genomic level.
Created in BioRender. Harris, A. (2024) https://BioRender.com/p55u051.

© 2025 Harris AR et al. *JAMA Network Open*.

eTable 1. Serum Proteomics-Defined Pathways Captured by the 92 Immune-Oncology Markers

Angiogenesis/Vascular	Metabolism/Autophagy	Suppression of Anti-tumor Immunity	Promotion of Anti-tumor Immunity	Apoptosis/Cell Killing	Chemotaxis
ADGRG1	ADA	ARG1	CD244	CASP-8	CCL17
ANG-1	CAIX	CCL17	CD27	CD40-L	CCL19
ANGPT2	HO-1	CCL19	CD28	FASLG	CCL20
CAIX		CCL20	CD40-L	GZMA	CCL23
CXCL1		Gal-9	CD40	GZMB	CCL3
CXCL10		CSF-1	CD5	GZMH	CCL4
CXCL5		CXCL1	CD70	MMP7	CX3CL1
CXCL9		CXCL5	CD83	TNFRSF12A	CXCL1
DCN		Gal-1	CD8A	TNFRSF21	CXCL10
EGF		IL10	CRTAM	TRAIL	CXCL11
FGF2		IL4	CX3CL1	TWEAK	CXCL13
Gal-1		IL5	CXCL10	Gal-9	CXCL5
HGF		IL8	CXCL11		CXCL9
IL12		LAMP3	CXCL13		IL8
IL8		LAP TGF-beta-1	CXCL9		MCP-1
MCP-1		MIC-A/B	ICOSLG		MCP-2
MMP12		MMP12	IL12RB1		MCP-3
NOS3		MMP7	IL18		MCP-4
PDGF subunit B		PD-L1	IL6		
PGF		PD-L2	IL7		
PTN		PDCD1	KLRD1		
TIE2			NCR1		
TNFRSF12A			TNFRSF4		
VEGFA			TNFRSF9		
VEGFC			TNFSF14		
VEGFR-2					
Gal-9					
CXCL11					
CCL23					
TWEAK					

Shown are the markers whose serum levels were used to calculate the pathway activity scores

© 2025 Harris AR et al. *JAMA Network Open*.

eTable 2. Correlations Between Multilevel Stress-Related Determinants

Participants, n=121	Stress [a]	Social Support [a]	Discrimination [b] (Black women only)	Neighborhood Deprivation
Stress [a]	**-**			
Social Support [a]	0.15	**-**		
Discrimination (Black women only)	0.24	**0.43****	-	
Neighborhood Deprivation	-0.08	0.14	0.02	**-**

[a] Perceived
[b] Composite variable including both perceptions of race-based discrimination and related emotional and physical symptoms.
** *P*<0.01

© 2025 Harris AR et al. *JAMA Network Open*.

eTable 3. Participant Characteristics Within the Proteomic Analytic Sample

	Overall	Stress [a]	Social Support [a]	Discrimination [b] (Black women only)		Neighborhood Deprivation
	n=117	Mean (SD)	Mean (SD)	No Experience	≥1 Experiences	Mean (SD)
**Age (mean, SD)**	56.26 (12.37)	-	-	57.08 (12.93)	54.16 (13.28)	-
**Race**	*n (%)*					
White	65 (55.56)	18.28 (6.91)	61.32 (4.52)	-	-	-0.22 (2.01)
Black	52 (44.44)	16.33 (8.46)	62.27 (5.30)	24 (55.81)	19 (44.19)	2.28 (2.30)
**BMI, kg/m2 (mean, SD)**	31.53 (7.04)	-	-	33.37 (7.31)	35.01 (5.12)	-
**SES (composite)**	***n (%)***					
High school degree or less, Below Poverty Threshold	27 (23.08)	17.11 (8.71)	64.67 (6.26)	7 (29.17)	6 (31.58)	2.78 (2.38)
More than high school degree, Below Poverty Threshold	21 (17.95)	20.33 (7.15)	60.81 (2.84)	7 (29.17)	5 (26.32)	1.78 (2.37)
High school degree or less, Above Poverty Threshold	15 (12.82)	17.13 (7.04)	60.04 (5.50)	2 (8.33)	3 (15.79)	0.77 (2.39)
More than high school degree, Above Poverty Threshold	51 (43.59)	16.08 (6.88)	61.02 (4.13)	7 (29.17)	5 (26.32)	-0.50 (1.63)
**Tumor stage**	***n (%)***					
0	7 (6.14)	21 (6.32)	61.29 (3.99)	1 (4.35)	0	0.10 (2.05)
1	42 (36.84)	14.98 (6.74)	61.55 (4.23)	6 (26.09)	7 (41.18)	1.04 (2.72)
2	46 (40.35)	17.91 (8.13)	62.48 (5.93)	13 (56.52)	6 (35.29)	0.84 (2.53)
3	19 (16.67)	20.05 (7.49)	60.47 (3.73)	3 (13.04)	4 (23.53)	1.00 (2.02)
**Tumor molecular subtype**	***n (%)***					
Luminal A	0	-	-	0	0	-
Luminal B	84 (74.34)	18.29 (7.65)	61.19 (4.11)	16 (69.57)	8 (47.06)	0.76 (2.64)
HER2-enriched	5 (4.42)	12.20 (3.56)	59.8 (7.12)	0	0	0.13 (1.24)
Triple-negative	24 (21.24)	16.04 (7.45)	63.75 (6.58)	7 (30.43)	9 (52.94)	1.62 (1.98)

[a] *Perceived*

[b] *Composite variable including both race-based discrimination and related emotional and physical symptoms*

© 2025 Harris AR et al. *JAMA Network Open*.

eTable 4. Participant Characteristics Within the Tumor Transcriptomic Analytic Sample

	**Overall**	**Stress [a]**	**Social Support [a]**	**Discrimination [b] (Black only)**		**Neighborhood Deprivation**
	**n=48**	Mean (SD)	Mean (SD)	No Experience	≥1 Experiences	Mean (SD)
**Age (mean, SD)**	56.33 (13.92)		-	59.31 (15.31)	57.75 (15.3)	-
**Race**	***n (%)***					
White	20 (41.67)	20.05 (5.60)	61.7 (5.52)	-	-	-0.48 (1.51)
Black	28 (58.33)	16.21 (9.58)	63.75 (6.65)	13 (61.90)	8 (38.10)	2.28 (2.15)
**BMI, kg/m2 (mean, SD)**	32.25 (7.48)	-	-	34.81 (7.95)	35.21 (6.02)	-
**SES (composite)**	***n (%)***					
High school degree or less, Below Poverty Threshold	15 (31.25)	16.87 (9.66)	66.47 (6.91)	5 (38.46)	5 (62.50)	2.52 (2.29)
More than high school degree, Below Poverty Threshold	9 (18.75)	21.22 (9.20)	60.78 (2.33)	4 (30.77)	1 (12.50)	0.68 (2.09)
High school degree or less, Above Poverty Threshold	7 (14.58)	19.29 (8.73)	61.57 (6.60)	0	1 (12.50)	0.18 (1.39)
More than high school degree, Above Poverty Threshold	16 (33.33)	16.19 (6.46)	60.69 (5.33)	4 (30.77)	1 (12.50)	0.26 (2.19)
**Tumor stage**	***n (%)***					
0	0	-	-	0	0	-
1	11 (22.92)	17.36 (7.05)	60.91 (5.26)	2 (15.38)	2 (25.00)	1.41 (2.74)
2	24 (50.00)	17.5 (9.53)	64.58 (6.92)	10 (76.92)	3 (37.50)	1.09 (2.23)
3	13 (27.08)	18.77 (7.32)	61.46 (5.04)	1 (7.69)	3 (37.50)	0.97 (2.36)
**Tumor molecular subtype**	*n (%)*					
Luminal A	0	-	-	0	0	-
Luminal B	34 (72.34)	18.71 (7.78)	62.15 (5.02)	7 (58.33)	4 (50.00)	0.95 (2.46)
HER2-enriched	1 (2.13)	10	52	0	0	1.56
Triple-negative	12 (25.53)	17.08 (9.37)	66.25 (7.99)	5 (41.67)	4 (50.00)	1.59 (2.20)

[a] *Perceived*
[b] *Composite variable including both race-based discrimination and related emotional and physical symptoms.*

© 2025 Harris AR et al. *JAMA Network Open*.

eTable 5. Association of Perceived Stress, Social Support, Discrimination, and Neighborhood Deprivation With Serum Proteomics-Defined Biological Pathways

	**Full Sample**	**Black**	**White**
	Beta [95% CI]	Beta [95% CI]	Beta [95% CI]
**Angiogenesis/Vascular**			
Stress [a]	**0.34 [-.0006, 0.67] ***	**0.48 [0.04, 0.93] ****	0.26 [-0.30, 0.81]
Social Support [a]	-0.01 [-0.51, 0.49]	0.12 [-0.57, 0.81]	-0.17 [-0.95, 0.61]
Racial and ethnic discrimination	N/A	2.25 [-5.71, 10.20]	N/A
Neighborhood Deprivation	-0.17 [-1.37, 1.03]	-0.17 [-1.79, 1.44]	-0.45 [-2.63, 1.73]
**Metabolism**			
Stress [a]	0.03 [-0.02, 0.08]	0.06 [-0.02, 0.13]	-0.01 [-0.08, 0.07]
Social Support [a]	0.003 [-0.08, 0.07]	-0.03 [-0.14, 0.08]	0.02 [-0.08, 0.13]
Racial and ethnic discrimination	N/A	0.38<br>[-0.94, 1.70]	N/A
Neighborhood Deprivation	-0.04 [-0.22, 0.14]	-0.01 [-0.28, 0.25]	-0.08 [-0.39, 0.24]
**Suppression of Anti-Tumor Immunity**			
Stress [a]	0.22 [-0.05, 0.49]	**0.37 [-0.002, 0.75] ***	0.06 [-0.37, 0.49]
Social Support [a]	-0.02 [-0.43, 0.38]	0.18 [-0.39, 0.75]	-0.30 [-0.90, 0.29]
Racial and ethnic discrimination		3.00 [-3.76, 9.76]	
Neighborhood Deprivation	0.07 [-0.91, 1.05]	0.03 [-1.32, 1.39]	-0.2 [-1.97, 1.50]
**Promotion of Anti-Tumor Immunity**			
Stress [a]	0.23 [-0.16, 0.62]	0.30 [-0.24, 0.84]	0.12 [-0.49, 0.73]
Social Support [a]	-.005 [-0.58, 0.57]	0.19 [-0.62, 0.99]	-0.36 [-1.21, 0.49]
Racial and ethnic discrimination		4.72 [-5.05, 14.50]	
Neighborhood Deprivation	0.30 [-1.09, 1.68]	-0.34 [-2.25, 1.57]	0.13 [-2.31, 2.57]

© 2025 Harris AR et al. *JAMA Network Open*.

**Apoptosis/Cell Death**			
Stress [a]	-0.01 [-0.18, 0.17]	0.04 [-0.18, 0.27]	-0.08 [-0.38, 0.22]
Social Support [a]	-0.06 [-0.32, 0.2]	0.19 [-0.14, 0.52]	**-0.36 [-0.77, 0.05] ***
Racial and ethnic discrimination		0.53 [-3.26, 4.32]	
Neighborhood Deprivation	0.34 [-0.29, 0.97]	0.51 [-0.27, 1.29]	-0.25 [-1.46, 0.95]
**Chemotaxis**			
Stress [a]	**0.22 [-0.01, 0.46] ***	**0.28 [0.001, 0.56] ****	0.18 [-0.23, 0.60]
Social Support [a]	0.05 [-0.31, 0.4]	0.13 [-0.29, 0.56]	-0.08 [-0.66, 0.50]
Racial and ethnic discrimination		1.19 [-3.82, 6.19]	
Neighborhood Deprivation	-0.47 [-1.31, 0.37]	-0.64 [-1.59, 0.32]	-0.57 [-2.22, 1.09]

Separate models adjusted for age at study entry (continuous), body mass index at study entry (continuous), self-reported race (Black, White; not included in racial and ethnic discrimination model) and socio-economic status [i.e., 1) high school or less and in poverty, 2) at least some college and in poverty, 3) high school or less and not in poverty, 4) at least some college and not in poverty]. Poverty was defined using income and federally defined poverty threshold based on household size, age, and recruitment year. Perceived stress and social support models conducted in full sample, n = 117, Black n = 52, White n = 65. Neighborhood deprivation model conducted in full sample n = 111, Black or Hispanic/Latina n = 50, White n = 61. Racial and discrimination model conducted only among Black, n = 43.

* $P < 0.10$, ** $P < 0.05$.

[a] Perceived.

© 2025 Harris AR et al. *JAMA Network Open*.

eTable 6. Associations Between Multilevel Stress-Related Determinants and Serum Proteomics-Defined Pathways Adjusted for Individual, Interpersonal, and Neighborhood-Level Factors

	**Full Sample**	
	Model 1	Model 2
	**With individual and interpersonal characteristics, n = 117**	**Model 1 + neighborhood deprivation, n = 111**
	Beta [95% CI]	Beta [95% CI]
**Angiogenesis/Vascular**		
Stress [a]	**0.35 [0.01, 0.70] ****	0.24 [-0.11, 0.60]
Social Support [a]	-0.12 [-0.63, 0.39]	-0.13 [-0.63, 0.38]
Neighborhood Deprivation		-0.11 [-1.32, 1.09]
**Metabolism**		
Stress [a]	0.03 [-0.02, 0.08]	0.02 [-0.03, 0.08]
Social Support [a]	-0.01 [-0.09, 0.06]	-0.01 [-0.08, 0.07]
Neighborhood Deprivation		-0.03 [-0.22, 0.15]
**Suppression of Anti-Tumor Immunity**		
Stress [a]	**0.24 [-0.04, 0.52] ***	0.17 [-0.12, 0.46]
Social Support [a]	-0.1 [-0.51, 0.31]	-0.10 [-0.51, 0.31]
Neighborhood Deprivation		0.11 [-0.88, 1.1]
**Promotion of Anti-Tumor Immunity**		
Stress [a]	0.25 [-0.15, 0.65]	0.15 [-0.26, 0.57]
Social Support [a]	-0.08 [-0.67, 0.50]	-0.09 [-0.68, 0.49]
Neighborhood Deprivation		0.33 [-1.07, 1.73]

© 2025 Harris AR et al. *JAMA Network Open*.

**Apoptosis/Cell Death**		
Stress [a]	0.004 [-0.18, 0.19]	-0.05 [-0.24, 0.14]
Social Support [a]	-0.06 [-0.33, 0.21]	-0.06 [-0.33, 0.21]
Neighborhood Deprivation		0.32 [-0.32, 0.96]
**Chemotaxis**		
Stress [a]	**0.23 [-0.02, 0.47] ***	0.14 [-0.11, 0.39]
Social Support [a]	-0.03 [-0.38, 0.33]	-0.04 [-0.39, 0.32]
Neighborhood Deprivation		-0.43 [-1.28, 0.41]

Models adjusted for age at study entry (continuous), body mass index at study entry (continuous), self-reported race (Black, White) and socio-economic status [i.e., 1) high school or less and in poverty, 2) at least some college and in poverty, 3) high school or less and not in poverty, 4) at least some college and not in poverty]. Poverty was defined using income and federally defined poverty threshold based on household size, age, and recruitment year.
* $P < 0.1$, ** $P < 0.05$
[a] Perceived.

© 2025 Harris AR et al. *JAMA Network Open*.

eTable 7. Differential Gene Expression and Pathway Enrichment Analyses From Upregulated Genes in Relation to Multilevel Stress-Related Determinants in Tumor and Tumor-Adjacent Normal Tissues

**Stress [a]**							
**Top Differentially Over-Expressed Genes [c]**							
Number of Differentially Expressed Genes (FDR<0.1): ***68***							
**Normal** (DEGs: 17; Up: 8; Down: 9)				**Tumor** (DEGs: 51; Up: 20; Down: 31)			
**Gene Symbol**	**Gene Name**	**Log2 FC [d]**	**FDR**	**Gene Symbol**	**Gene Name**	**Log2 FC [d]**	**FDR**
CCL18	C-C Motif Chemokine Ligand 18	2.71	6.36E-02	TBC1D3E	TBC1 Domain Family Member 3E	7.05	3.48E-02
F8A3	Coagulation Factor VIII Associated 3	1.67	1.36E-04	TRPM8	Transient Receptor Potential Cation Channel Subfamily M Member 8	5.86	3.27E-03
DNAH10	Dynein Axonemal Heavy Chain 10	1.57	9.98E-02	ABCA13	ATP Binding Cassette Subfamily A Member 13	4.49	1.72E-02
				HLA-G	Major Histocompatibility Complex, Class I, G	4.18	1.72E-02
				CR2	Complement C3d Receptor 2	4.04	4.15E-02
Shared DEGs: *3*							
**Significantly Enriched Pathways in Tumor -** ***Upregulated***							
Number of Significantly Enriched Pathways - Upregulated (Adj. P < 0.1): ***6***							

**Pathway**	**Adj. P-Value**
Dopamine Receptors	9.31E-02
RUNX1 regulates transcription of genes involved in BCR signaling	9.31E-02
Immunoregulatory interactions between a lymphoid and a non-lymphoid cell	9.31E-02
Immune system	9.31E-02
Endosomal/Vacuolar pathway	9.31E-02
Interleukin-27 signaling	9.31E-02

**Social Support [a]**							
**Top Differentially Over-Expressed Genes [c]**							
Number of Differentially Expressed Genes (FDR<0.1): ***54***							
**Normal** (DEGs: 16; Up: 12; Down: 4)				**Tumor** (DEGs: 38; Up: 22; Down: 16)			
**Gene Symbol**	**Gene Name**	**Log2 FC [d]**	**FDR**	**Gene Symbol**	**Gene Name**	**Log2 FC [d]**	**FDR**
IGLV3-19	Immunoglobulin Lambda Variable 3-19	3.55	9.75E-02	TBC1D3E	TBC1 Domain Family Member 3E	7.22	3.26E-02
MSLN	Mesothelin	3.37	9.29E-02	IGKV1-33	Immunoglobulin Kappa Variable 1-33	6.06	3.01E-03
ADAMDEC1	ADAM Like Decysin 1	3.04	9.75E-02	GJB5	Gap Junction Protein Beta 5	4.42	3.46E-04
CCL18	C-C Motif Chemokine Ligand 18	3.02	4.42E-02	LPO	Lactoperoxidase	4.20	3.26E-02
F8A3	Coagulation Factor VIII Associated 3	1.74	4.37E-05	HLA-G	Major Histocompatibility Complex, Class I, G	4.01	3.26E-02
Shared DEGs: *3*							
**Significantly Enriched Pathways in Tumor -** ***Upregulated***							
Number of Significantly Enriched Pathways - Upregulated (Adj. P < 0.1): ***0***							

**Discrimination [b]**							
**Top Differentially Over-Expressed Genes [c]**							
Number of Differentially Expressed Genes (FDR<0.1): ***902***							
**Normal** (DEGs: 295; Up: 52; Down: 243)				**Tumor** (DEGs: 607; Up: 270; Down: 337)			
**Gene Symbol**	**Gene Name**	**Log2 FC [d]**	**FDR**	**Gene Symbol**	**Gene Name**	**Log2 FC [d]**	**FDR**
CDH19	Cadherin 19	3.30	7.72E-02	ACADL	Acyl-CoA Dehydrogenase Long Chain	6.54	4.95E-03
SCD	Stearoyl-CoA Desaturase	2.88	1.55E-02	OBP2B	Odorant Binding Protein 2B	6.52	4.30E-03
SLC7A4	Solute Carrier Family 7 Member 4	2.55	8.01E-02	GDF5	Growth Differentiation Factor 5	6.49	1.62E-04
NNAT	Neuronatin	2.24	9.84E-02	CHIT1	Chitinase 1	6.47	2.23E-05
MGST1	Microsomal Glutathione S-Transferase 1	2.03	7.99E-02	SPX	Spexin Hormone	6.40	5.11E-03
Shared DEGs: *45*							
**Significantly Enriched Pathways in Tumor -** ***Upregulated***							
Number of Significantly Enriched Pathways - Upregulated (Adj. P < 0.1): ***15***							

**Pathway**	**Adj. P-Value**
Endosomal/Vacuolar pathway	1.64E-06
Interferon Gamma Response	1.68E-03
Allograft Rejection	1.68E-03
IL-6/JAK/STAT3 Signaling	2.17E-03
Hypoxia	3.74E-03
Antigen Presentation: folding, assembly, peptide loading of class I MHC	1.08E-02
Interferon-alpha/beta signaling	1.15E-02
Cytokine signaling in immune system	3.81E-02
Antigen processing-cross presentation	6.93E-02
TNF-alpha Signaling via NF-kB	1.21E-02
UV Response Up	3.14E-02

© 2025 Harris AR et al. *JAMA Network Open*.

Pathway				Adj. P-Value			
Complement				3.14E-02			
Bile Acid Metabolism				7.10E-02			
IL-2/STAT5 Signaling				7.10E-02			
KRAS Signaling Up				7.10E-02			
**Neighborhood Deprivation**							
**Top Differentially Over-Expressed Genes [c]**							
*Number of Differentially Expressed Genes (FDR<0.1): **33***							
**Normal** (DEGs: 11; Up: 6; Down: 5)				**Tumor** (DEGs: 22; Up: 11; Down: 11)			
**Gene Symbol**	**Gene Name**	**Log2 FC [d]**	**FDR**	**Gene Symbol**	**Gene Name**	**Log2 FC [d]**	**FDR**
CCL18	C-C Motif Chemokine Ligand 18	3.10	1.52E-05	GOLGA6B	Golgin A6 Family Member B	4.11	1.52E-02
DNAH10	Dynein Axonemal Heavy Chain 10	1.96	5.62E-06	GJB5	Gap Junction Protein Beta 5	3.82	9.48E-02
F8A3	Coagulation Factor VIII Associated 3	1.71	1.31E-03	MELTF	Melanotransferrin	2.14	9.85E-02
				F8A3	Coagulation Factor VIII Associated 3	2.05	1.92E-03
				RFXAP	Regulatory Factor X Associated Protein	0.78	9.85E-02
Shared DEGs: *3*							
**Significantly Enriched Pathways in Tumor - *Upregulated***							
*Number of Significantly Enriched Pathways - Upregulated (Adj. P < 0.1): **3***							
**Pathway**				**Adj. P-Value**			
Gap Junction Assembly				4.92E-02			
Gap Junction Trafficking				4.92E-02			
Gap Junction Trafficking and Regulation				4.92E-02			

All models adjusted for the following covariates: age at diagnosis, body mass index, socioeconomic status (composite), race (with exception of discrimination models in Black women only)

[a] Perceived

[b] Discrimination models subsetted to include only Black participants

[c] Top 5 significant (FDR<0.1) differentially upregulated protein-coding genes based on log2 fold change

[d] FC: Fold change

© 2025 Harris AR et al. *JAMA Network Open*.

eTable 8. Differential Gene Expression and Pathway Enrichment Analyses From Downregulated Genes in Relation to Multilevel Stress-Related Determinants in Tumor and Tumor-Adjacent Normal Tissues

**Stress [a]**							
**Top Differentially Under-Expressed Genes [c]**							
Number of Differentially Expressed Genes (FDR<0.1): ***68***							
**Normal** (DEGs: 17; Up: 8; Down: 9)				**Tumor** (DEGs: 51; Up: 20; Down: 31)			
**Gene Symbol**	**Gene Name**	**Log2 FC [d]**	**FDR**	**Gene Symbol**	**Gene Name**	**Log2 FC**	**FDR**
FSIP2	Fibrous Sheath Interacting Protein 2	-1.74	8.08E-02	NSG2	Neuronal Vesicle Trafficking Associated 2	-3.90	3.55E-02
SERHL2	Serine Hydrolase Like 2	-2.87	4.83E-02	CDH7	Cadherin 7	-3.62	2.60E-02
LEKR1	Leucine, Glutamate And Lysine Rich 1	-1.29	6.16E-02	SCGB3A1	Secretoglobin Family 3A Member 1	-3.34	7.87E-02
FBXO25	F-Box Protein 25	-0.50	4.14E-02	CHST8	Carbohydrate Sulfotransferase 8	-3.05	5.78E-02
				CLIC6	Chloride Intracellular Channel 6	-3.03	3.48E-02
Shared DEGs: *3*							
**Significantly Enriched Pathways in Tumor - *Downregulated***							
Number of Significantly Enriched Pathways - Downregulated (Adj. P < 0.1): ***0***							
**Social Support [a]**							
**Top Differentially Under-Expressed Genes [c]**							
Number of Differentially Expressed Genes (FDR<0.1): ***54***							
**Normal** (DEGs: 16; Up: 12; Down: 4)				**Tumor** (DEGs: 38; Up: 22; Down: 16)			
**Gene Symbol**	**Gene Name**	**Log2 FC**	**FDR**	**Gene Symbol**	**Gene Name**	**Log2 FC**	**FDR**
FBXO25	F-Box Protein 25	-0.50	9.23E-02	CLIC6	Chloride Intracellular Channel 6	-3.09	3.26E-02
				SLC1A1	Solute Carrier Family 1 Member 1	-2.72	8.48E-02
				THPO	Thrombopoietin	-2.45	4.53E-02
				CCDC144A	Coiled-Coil Domain Containing 144A	-2.21	9.79E-02
				IL20RA	Interleukin 20 Receptor Subunit Alpha	-2.02	6.64E-02
Shared DEGs: *3*							
**Significantly Enriched Pathways in Tumor - *Downregulated***							
Number of Significantly Enriched Pathways - Downregulated (Adj. P < 0.1): ***0***							
**Discrimination [b]**							
**Top Differentially Under-Expressed Genes [c]**							
Number of Differentially Expressed Genes (FDR<0.1): ***902***							
**Normal** (DEGs: 295; Up: 52; Down: 243)				**Tumor** (DEGs: 607; Up: 270; Down: 337)			
**Gene Symbol**	**Gene Name**	**Log2 FC**	**FDR**	**Gene Symbol**	**Gene Name**	**Log2 FC**	**FDR**
TRIM6-TRIM34	Tripartite Motif-Containing 6 And Tripartite Motif-Containing 34	-5.14	7.48E-02	POTEC	POTE Ankyrin Domain Family Member C	-9.79	1.35E-02
ZNF556	Zinc Finger Protein 556	-5.06	3.48E-02	SRARP	Steroid Receptor Associated and Regulated Protein	-8.16	3.01E-02
KLK3	Kallikrein Related Peptidase 3	-4.95	6.64E-02	ASCL1	Achaete-Scute Family BHLH Transcription Factor 1	-7.29	3.75E-02
GRIK1	Glutamate Ionotropic Receptor Kainate Type Subunit 1	-4.59	6.64E-02	AGR2	Anterior Gradient 2, Protein Disulphide Isomerase Family Member	-6.80	5.38E-07
POU3F3	POU Class 3 Homeobox 3	-4.27	3.88E-02	AGR3	Anterior Gradient 3, Protein Disulphide Isomerase Family Member	-6.62	1.69E-03
Shared DEGs: *45*							
**Significantly Enriched Pathways in Tumor - *Downregulated***							
Number of Significantly Enriched Pathways - Downregulated (Adj. P < 0.1): ***10***							
**Pathway**				**Adj. P-Value**			
Estrogen response - Early				1.09E-15			
Estrogen response - Late				8.45E-09			
KRAS signaling - Down				7.41E-04			

© 2025 Harris AR et al. *JAMA Network Open*.

Signaling by nuclear receptors	2.90E-03
ESR mediated signaling	7.60E-03
Cardiac conduction	9.32E-03
Degradation of the extracellular matrix	1.28E-02
Estrogen dependent gene expression	1.57E-02
Muscle contraction	1.57E-02
Phase 2 plateau phase	2.34E-02

Neighborhood Deprivation

Top Differentially Under-Expressed Genes [c]

Number of Differentially Expressed Genes (FDR<0.1): ***33***

**Normal** (DEGs: 11; Up: 6; Down: 5)				**Tumor** (DEGs: 22; Up: 11; Down: 11)			
**Gene Symbol**	**Gene Name**	**Log2 FC**	**FDR**	**Gene Symbol**	**Gene Name**	**Log2 FC**	**FDR**
ABCC11	ATP Binding Cassette Subfamily C Member 11	-3.18	7.93E-02	TMC3	Transmembrane Channel Like 3	-5.28	9.91E-02
				CCDC39	Coiled-Coil Domain 39 Molecular Ruler Complex Subunit	-2.02	9.85E-02
				GPAT2	Glycerol-3-Phosphate Acyltransferase 2, Mitochondrial	-1.79	1.81E-02
				MON2	MON2 Homolog, Regulator Of Endosome-To-Golgi Trafficking	-0.59	1.52E-02

Shared DEGs: *3*

Significantly Enriched Pathways in Tumor - *Downregulated*

Number of Significantly Enriched Pathways - Downregulated (Adj. P < 0.1): ***0***

All models adjusted for the following covariates: age at diagnosis, body mass index, socioeconomic status (composite), race (with exception of discrimination models in Black women only)

[a] Perceived

[b] Discrimination models subsetted to include only Black participants

[c] Top 5 significant (FDR<0.1) differentially down-regulated protein-coding genes based on log2 fold change

[d] FC: Fold change

© 2025 Harris AR et al. *JAMA Network Open*.
