# Supplementary material for: Multilevel Stressors and Systemic and Tumor Immunity in Black and White Women With Breast Cancer
Source: JAMA Netw Open. 2025 Feb 14;8(2):e2459754. doi: 10.1001/jamanetworkopen.2024.59754 (PMC11829235; doi:10.1001/jamanetworkopen.2024.59754)
Supplement: Supplement 3. — Data Sharing Statement [file jamanetwopen-e2459754-s003.pdf]

Data Sharing Statement

Harris. Multilevel Stressors and Systemic and Tumor Immunity in Black and White Women With Breast Cancer. *JAMA Netw Open*. Published February 14, 2025. doi:10.1001/jamanetworkopen.2024.59754

Data
Data available: Yes
Data types: Deidentified participant data
How to access data: The RNA sequencing (RNA-seq) data were deposited in the NCBI's Gene Expression Omnibus (GEO) database under accession number GSE225846 and GSE267505. Whole exome sequencing data were deposited in the NCBI SRA database under the accession number PRJNA1111785 and PRJNA913947. To review GEO accession GSE267505: Go to https://www.ncbi.nlm.nih.gov/geo/query/acc.cgi?acc=GSE267505 Enter token oberswyopzotboz into the box BioProject and associated SRA metadata are available at https://dataview.ncbi.nlm.nih.gov/object/PRJNA1111785?reviewer=9ni05ls7t9m5nkmdn0k5snf7qr in read-only format.
When available: With publication

Supporting Documents
Document types: None

Additional Information
Who can access the data: Publicly available
Types of analyses: For any purpose
Mechanisms of data availability: Without investigator support
Any additional restrictions: None
